# PPARG Activation of Fatty Acid Metabolism Drives Resistance to Anti-HER2 Therapies in HER2-Positive Breast Cancer

**DOI:** 10.7150/ijbs.99275

**Published:** 2025-03-10

**Authors:** Min Xiong, Douwaner Liu, Xuliren Wang, Hengyu Ren, Weiru Chi, Bingqiu Xiu, Qi Zhang, Jiayu Zhang, Liyi Zhang, Zehao Wang, Ming Chen, Jingyan Xue, Yayun Chi, Jiong Wu

**Affiliations:** 1 Key Laboratory of Breast Cancer in Shanghai, Department of Breast Surgery, Fudan University Shanghai Cancer Center, Shanghai, 200032, China.; 2 Department of Oncology, Shanghai Medical College, Fudan University, Shanghai, 200032, China.; 3 Tianjin Medical University Cancer Institute and Hospital, Tianjin, 300060, China.; 4 Pathology Center, Shanghai General Hospital, Shanghai Jiao Tong University School of Medicine, Shanghai, China.

**Keywords:** HER2-positive breast cancer, PPARG, GW9662, Drug sensitivity

## Abstract

HER2-positive breast cancer, which accounts for approximately 15-20% of all breast cancers, is characterized by its aggressive recurrence, metastasis and reduced survival. Despite advances in anti-HER2 therapies, many patients continue to face treatment resistance, either initially or after an initial positive response, resulting in relapse or disease progression. The primary focus of this research was to identify the peroxisome proliferator-activated receptor gamma (PPARG) as a contributing factor to decreased drug sensitivity by establishing anti-HER2 drug-resistant cell lines of HER2-positive breast cancer. We found that PPARG promotes fatty acid metabolism and activates the PI3K/Akt/mTOR signaling pathway. Inhibition of fatty acid synthesis (FASN) after overexpression of PPARG, effectively blocking the activation of the PI3K/Akt/mTOR pathway and enhancing cellular anti-HER2 drug sensitivity. Co-administration of the PPARG inhibitor GW9662 has emerged as a promising strategy to augment the efficacy of anti-HER2 therapies, offering potential for clinical applications.

## Introduction

The latest epidemiological data on cancer revealed that in 2022, breast cancer ranked second in the world in terms of newly diagnosed cancers, while it was the leading cancer among females^1^. The human epidermal growth factor receptor (HER) constitutes a cluster of protein receptors endowed with tyrosine kinase activity, encompassing HER1, HER2, HER3, and HER4. Among these, the status of HER2 plays a crucial role in the initiation, progression, metastasis and prognosis of breast cancer^2^. Through the formation of heterodimers or homodimers, HER2 is capable of initiating a plethora of downstream signaling pathways, notably including the PI3K/Akt/mTOR pathway^3^, thereby driving malignant cell proliferation and contributing to drug resistance. According to the guidelines set forth by the American Society of Clinical Oncology-College of American Pathologists (ASCO-CAP), HER2 positivity is defined as tumors that exhibit 3+ positive staining by immunohistochemistry (IHC) in at least 10% of tumor cells, or detection of HER2 gene amplification through fluorescence in situ hybridization (FISH)^4^.

Approximately 15% of patients with early-stage HER2-positive breast cancer experience a recurrence after adjuvant treatment with trastuzumab, and patients with advanced HER2-positive breast cancer who are treated palliatively with trastuzumab tend to show disease progression within one year^5^. Despite the significant benefits brought by the neoadjuvant treatment regimen combining trastuzumab and pertuzumab (HP) for HER2-positive breast cancer patients^6^, there remains a proportion of 30% to 60% who fail to achieve pathological complete response (pCR) following neoadjuvant chemotherapy combined with HP^6^-^8^. Second-line use of trastuzumab emtansine (T-DM1) or tyrosine kinase inhibitors (TKIs) can also reverse the progression of the disease in some patients after the failure of first-line therapy, however there are still many patients for whom treatment with existing targeted therapies has not shown effective efficacy. Drug resistance in cancer cells is a critical factor in tumor progression, recurrence, and even mortality. In light of recent research, two key strategies have emerged in the field of fighting resistance to HER2-targeted therapies. One focuses on innovating drugs with novel structures and enhanced efficacy, while the other focuses on exploring combinations of drugs to effectively address resistance^9^.

The* in-vitro* establishment of drug-resistant cell lines serves as a valuable tool for studying the development of tumor resistance^10^. Various methods have been used to generate drug-resistant cell lines, including gene transfection and drug screening techniques. The latter encompasses strategies such as the high-dose drug intermittent pulse method, the drug concentration increase induction method, and a combination of both^11^. Prolonged exposure of cancer cells to drugs facilitates their adaptation and ultimately leads to the development of drug resistance^12^. The half maximal inhibitory concentration (IC_50_), which represents the concentration of a drug that inhibits cell growth by 50%, serves as a crucial parameter for evaluating sensitivity of cells to drugs. The resistance index (RI), defined as the ratio of IC_50_ for the drug-resistant cell line to the IC_50_ for the wild type cell line, exceeding 5 indicates successful construction of a drug-resistant cell line^13^,^14^.

This research identified the key gene PPARG, which plays a role in promoting resistance in HER2-positive breast cancer, through the construction of resistant cell lines and screening of public databases. PPARG is known for its high expression in adipose tissue and its involvement in various physiological processes such as fat synthesis, lipid storage and glucose regulation^15^. There is a lack of consensus on whether PPARG acts as a tumor suppressor or a promoter in cancer. Several studies have suggested that PPARG inhibits cyclins and CDKs, leading to cell cycle arrest and inhibition of tumor cell proliferation^16^, while others have indicated its role in maintaining stem cell viability and its association with brain metastasis in HER2-positive breast cancer^17^,^18^. Our research aims to identify the key gene that affects drug response in HER2-positive breast cancer, explore its specific mechanism in terms of drug sensitivity, and introduce specific inhibitor to investigate their potential clinical significance. We seek to contribute valuable insights for the development of more effective therapies for HER2-positive breast cancer.

## Results

### PPARG as a key factor in modulating drug sensitivity in HER2-positive breast cancer

**Figure 1:**
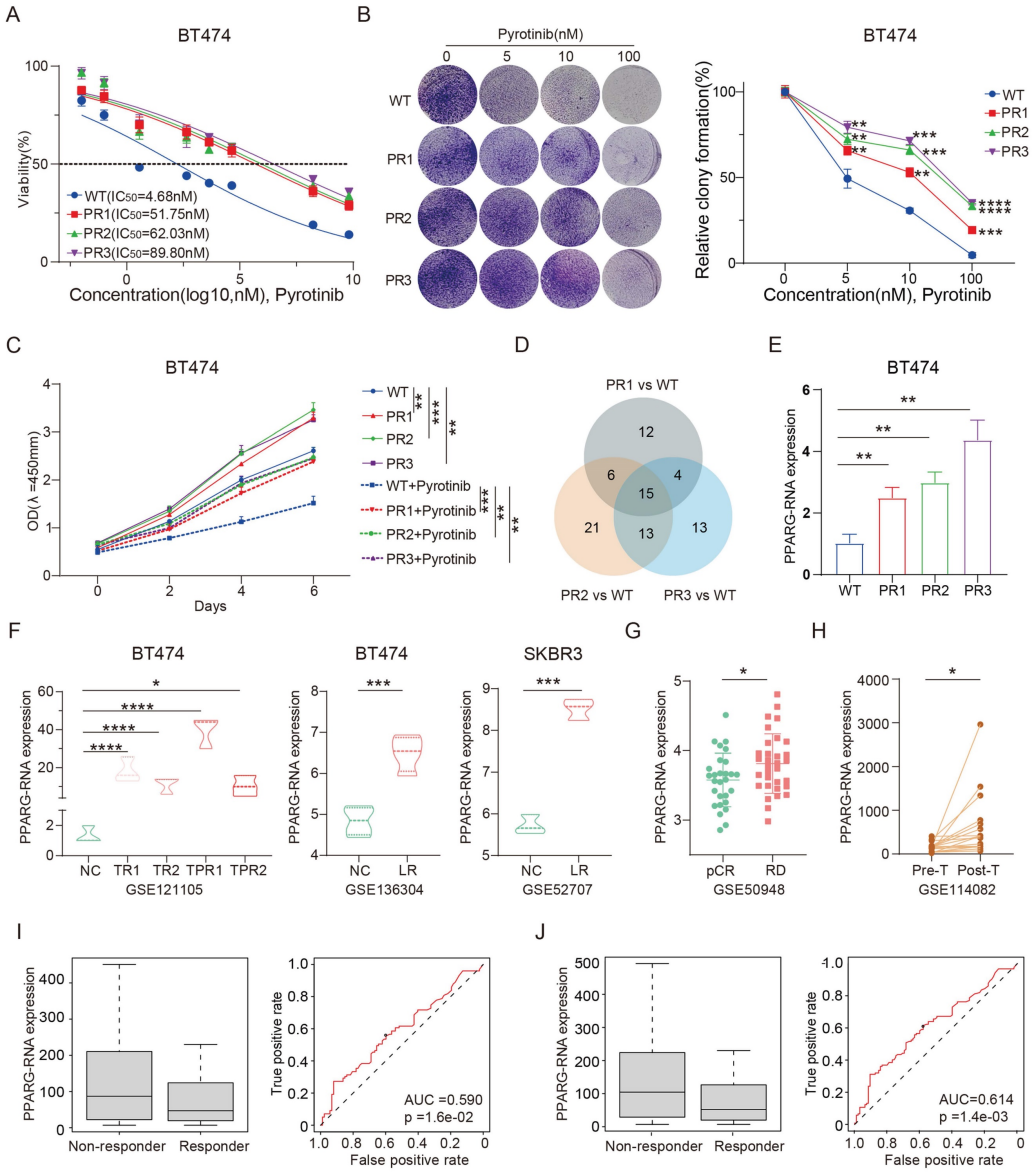
** PPARG as a key factor in modulating drug sensitivity in HER2-positive breast cancer.** (A) IC_50_ curves of WT and PR BT474 cells treated with gradient concentrations of pyrotinib; (B) Plate colony formation of WT and PR BT474 cells treated with different concentration pyrotinib; (C) Proliferation curves of WT and PR BT474 cells treated with or without pyrotinib; (D) Venn diagram illustrating elevated gene expression (ratio>2.5) in PR BT474 cells; (E) Expression profile of PPARG in WT and PR BT474 cells; (F) Expression profile of PPARG in BT474 cells from the GSE121105, GSE136304 and GSE52707 dataset; (G) Expression levels of PPARG in tumors of patients with pCR or RD from the GSE50948 dataset; (H) Expression levels of PPARG before and after trastuzumab treatment (paired T-test) from the GSE114082 dataset; Expression levels of PPARG and ROC curve analysis in non-responder and responder groups to (I) anti-HER2 therapy and (J) trastuzumab therapy. WT: Wild Type; PR: Pyrotinib-Resistant; NC: Normal Control; TR: Trastuzumab-Resistant; TPR: Resistant to both Trastuzumab and Pertuzumab; LR: Lapatinib-Resistant; pCR: pathological complete response; RD: residual disease.

We successfully derived three pyrotinib-resistant (PR) BT474 cell lines through the implementation of a high-dose drug intermittent pulse method upon meticulous validation, these cell lines demonstrated robust resistance to pyrotinib (**Figure 1A**), with the IC_50_ value of BT474 PR cells increasing more than tenfold compared to the wild-type (WT). Plate colony formation also showed that PR cell lines formed more colonies than the WT cells under continuous exposure to pyrotinib (**Figure 1B**). In addition, the PR cell lines exhibited enhanced proliferative capacity with a faster growth rate compared to the WT cells after with or without pyrotinib treatment (**Figure 1C**). These results collectively confirm the successful establishment of the reproducible pyrotinib-resistant cell lines. Subsequent genomic sequencing revealed that 15 genes were upregulated by at least a factor of 2.5 across all three resistant cell lines (**Figure 1D, Supplementary table 1**). Intriguingly, a concomitant elevation in the expression of the gene PPARG was observed alongside the increase in IC_50_ values in the cell line (**Figure 1E**). In addition, we meticulously gathered three distinct datasets from the publicly available GEO database: (1) GSE121105, which encompasses the BT474 wild-type, trastuzumab-resistant, and trastuzumab/pertuzumab-resistant cell lines; (2) GSE136304 consists of the BT474 wild-type and lapatinib resistant cell lines; (3) GSE52707 features the SKBR3 wild-type and lapatinib resistant cell lines. It is important to note that all three data sets consistently highlight enhanced expression of PPARG in resistant cell lines (**Figure 1F**).

Additionally, the GSE50948 dataset, consisting of sequencing data from patients in the NOAH clinical trial, showed an increase in PPARG expression levels in those with residual disease (RD) after neoadjuvant chemotherapy and anti-HER2 therapy (**Figure 1G**). Similarly, the GSE114082 dataset suggests a significant increase in PPARG expression levels in HER2-positive breast tumors following treatment with trastuzumab (**Figure 1H**). Analysis of the ROC Plotter database revealed that patients who did not respond to HER2 treatment had higher levels of PPARG expression (*p*=1.6e-02, AUC=0.590) (**Figure 1I**), particularly in those who did not respond to trastuzumab treatment (*p*=1.4e-03, AUC=0.614) (**Figure 1J**). The above findings suggest that up-regulation of PPARG is associated with resistance to anti-HER2 therapy.

### PPARG promotes resistance to anti-HER2 therapy in HER2-positive breast cancer

**Figure 2:**
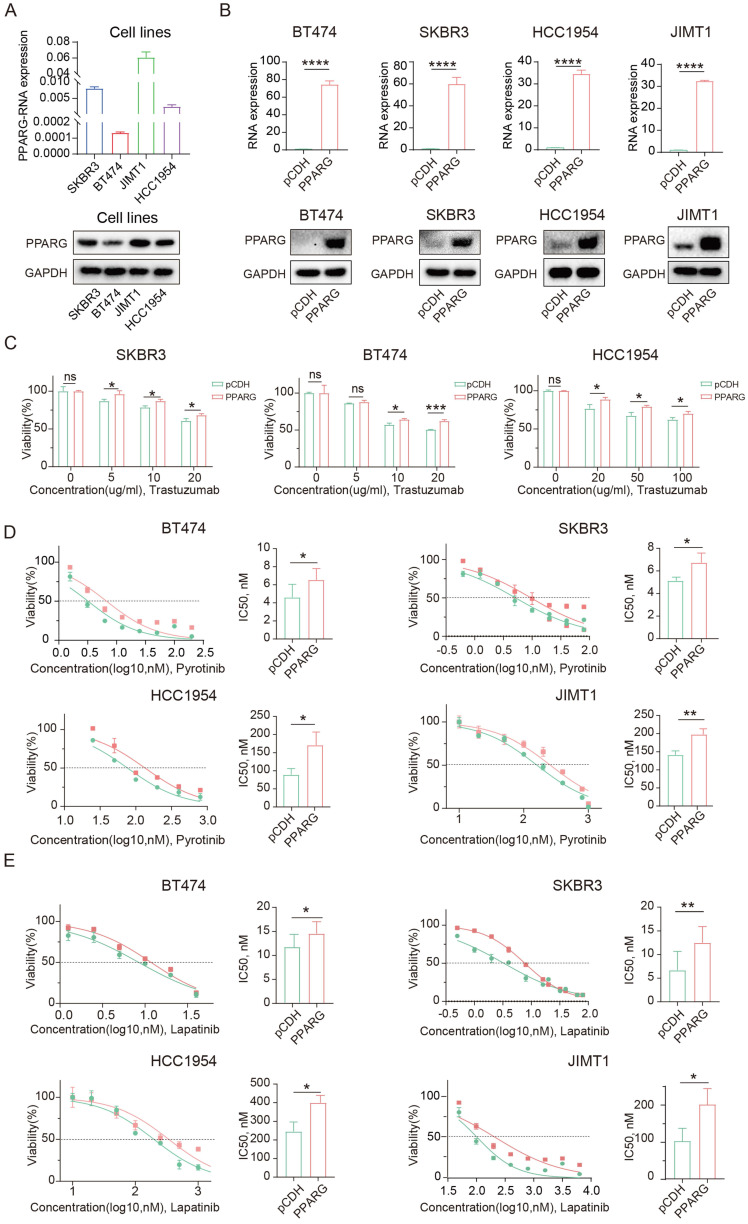
** PPARG promotes resistance to targeted therapy in HER2-positive breast cancer.** (A) Expression of PPARG at the RNA and protein level in four HER2-positive breast cancer cell lines (SKBR3, BT474, JIMT1, HCC1954 cells); (B) Expression of PPARG at the RNA and protein level in BT474, SKBR3, HCC1954, JIMT1 cells in pCDH and PPARG overexpressing groups; (C) Viability of different concentrations of trastuzumab treatment BT474, SKBR3, HCC1954 cells overexpressing PPARG compared to pCDH groups, (D) Gradient concentrations of pyrotinib treatment BT474, SKBR3, HCC1954, JIMT1 cells overexpressing PPARG compared to pCDH groups were plotted for IC_50_ curves and IC_50_ value bar graphs; (E) Gradient concentrations of lapatinib treatment BT474, SKBR3, HCC1954, JIMT1 cells overexpressing PPARG compared to pCDH groups were plotted for IC_50_ curves and IC_50_ value bar graphs.

We collected cell samples from four HER2-positive breast cancer cell lines to assess PPARG expression levels (**Figure 2A**), which showed high levels of PPARG in JIMT1 cells and low levels in BT474 cells. The JIMT1 cell is resistant to trastuzumab because it's derived from a 62-year-old patient with pleural metastatic breast cancer^19^. Based on these results, we can again demonstrate that PPARG is highly expressed in anti-HER2 resistant cells. Subsequently, we established PPARG-overexpressing cell lines in BT474, SKBR3, HCC1954, and JIMT1 cells (**Figure 2B**), and PPARG knockout cell lines in SKBR3, HCC1954 and JIMT1 cells (**Supplementary figure 1A**). Following PPARG overexpression, HER2-positive breast cancer cells showed reduced sensitivity to trastuzumab (**Figure 2C**), pyrotinib (**Figure 2D**), and lapatinib (**Figure 2E**). In turn, upon PPARG gene knockout, HER2-positive breast cancer cells showed enhanced sensitivity to anti-HER2 drugs (**Supplementary figure 1B, C, D**).

After combining trastuzumab and pyrotinib to treat HER2-positive breast cancer cells, cell survival was higher in the group with excess PPARG expression (**Supplementary figure 1E**), suggesting that PPARG may enhance resistance to combination therapy with dual-target drugs in HER2-positive breast cancer cells. Similarly, PPARG can counteract the combined cytotoxic effects of trastuzumab and lapatinib on HER2-positive breast cancer cells (**Supplementary figure 1F**).

### PPARG associated with poorer prognosis in HER2-positive breast cancer

**Figure 3:**
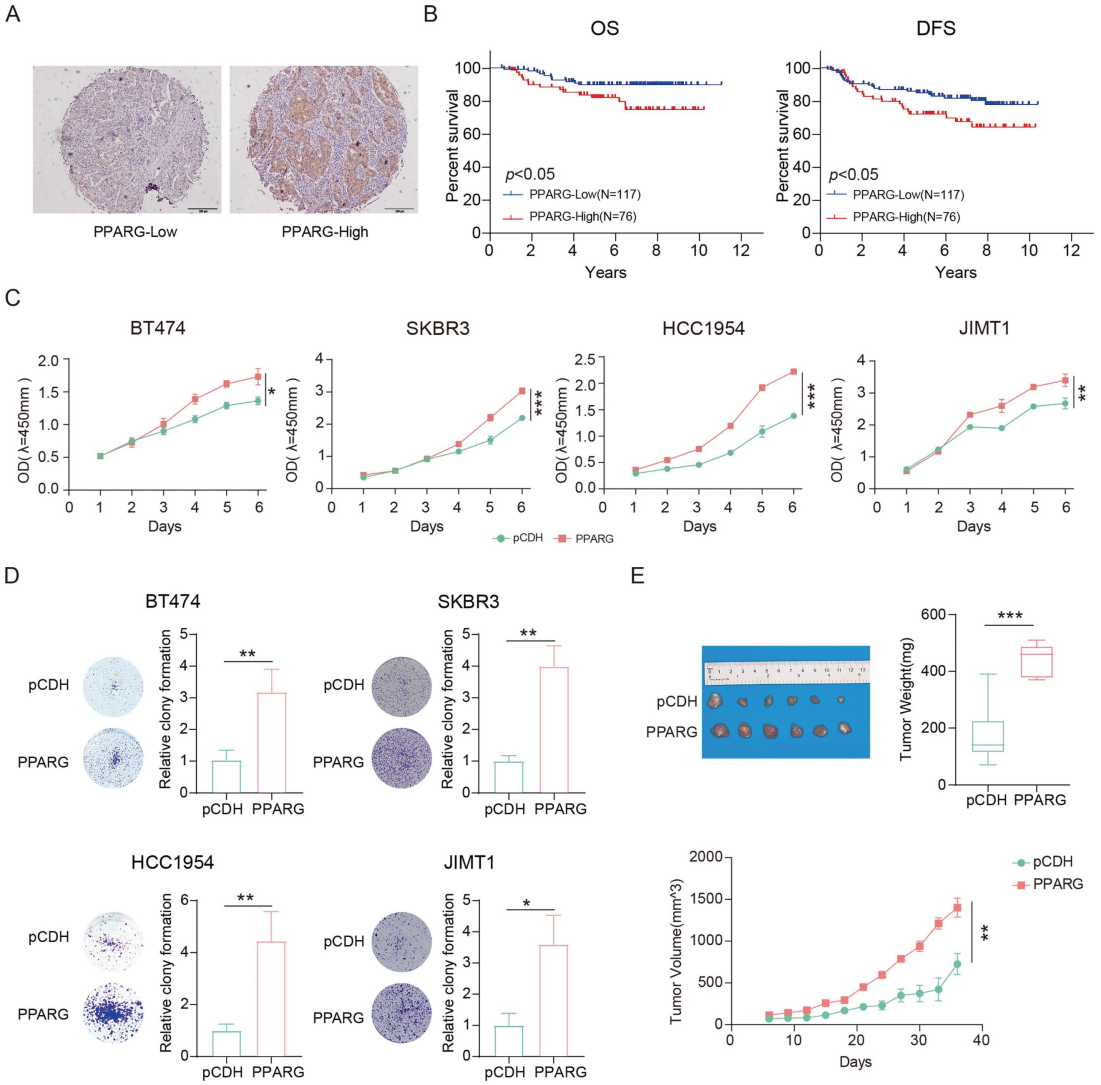
** PPARG associated with poorer prognosis in HER2-positive breast cancer.** (A) Examples of low and high expression of PPARG in tumor tissues; (B) Kaplan-Meier analysis of the relationship between PPARG expression levels and OS, DFS; (C) Proliferation curves of BT474, SKBR3, HCC1954, JIMT1 cells overexpressing PPARG compared to pCDH groups; (D) Plate colony formation of BT474, SKBR3, HCC1954, JIMT1 cells overexpressing PPARG compared to pCDH groups; (E) Schematic representation, mass box diagram and proliferation curves of in situ tumors in overexpressing PPARG compared to pCDH groups.

We performed immunohistochemical staining on tissue samples from 193 HER2-positive breast cancer patients and classified them into low and high expression groups based on the color rendering results of the PPARG antibody (**Figure 3A**). Our findings suggest that there is no correlation between PPARG expression and various clinical characteristics (**Supplementary table 2**). Univariate COX regression analysis showed that in HER2-positive breast cancer patients, OS correlated with PPARG expression level, histological grade, tumor size, and lymph node metastasis status (**Supplementary table 3**). Disease-free survival (DFS) was associated with lymph node metastasis status and PPARG expression level (**Supplementary table 4**). In the multivariate analysis, both OS and DFS were independently influenced by lymph node metastasis status and PPARG expression level in HER2-positive breast cancer patients (**Table 1, 2**).

Based on the clinical prognostic data of the patients from the tissue samples, Kaplan-Meier analysis indicated poorer OS and DFS in patients with high PPARG expression (**Figure 3B**). Molecular Taxonomy of Breast Cancer International Consortium (METABRIC) database collects sequencing data and survival data for patients with different subtypes of breast cancer. We classified HER2-positive breast cancer patients into two groups based on the median expression level of PPARG: high expression group and low expression group. Combining with survival data, we have also found that high expression of PPARG is associated with worse outcomes in patients (**Supplementary figure 2A, B**).

Overexpression of PPARG was found to enhance the proliferation of HER2-positive breast cancer cells (**Figure 3C**), while inhibition of PPARG resulted in inhibition of HER2-positive breast cancer cell proliferation (**Supplementary figure 2C**). Colony formation assays consistently confirm the role of PPARG in promoting the proliferation of HER2-positive breast cancer cells (**Figure 3D, Supplementary figure 2D**). It has also been shown that PPARG can indeed promote *in vivo* HER2-positive breast cancer proliferation (**Figure 3E**).

### Fatty acid metabolism is more active in anti-HER2 resistant HER2-positive breast cancer

**Figure 4:**
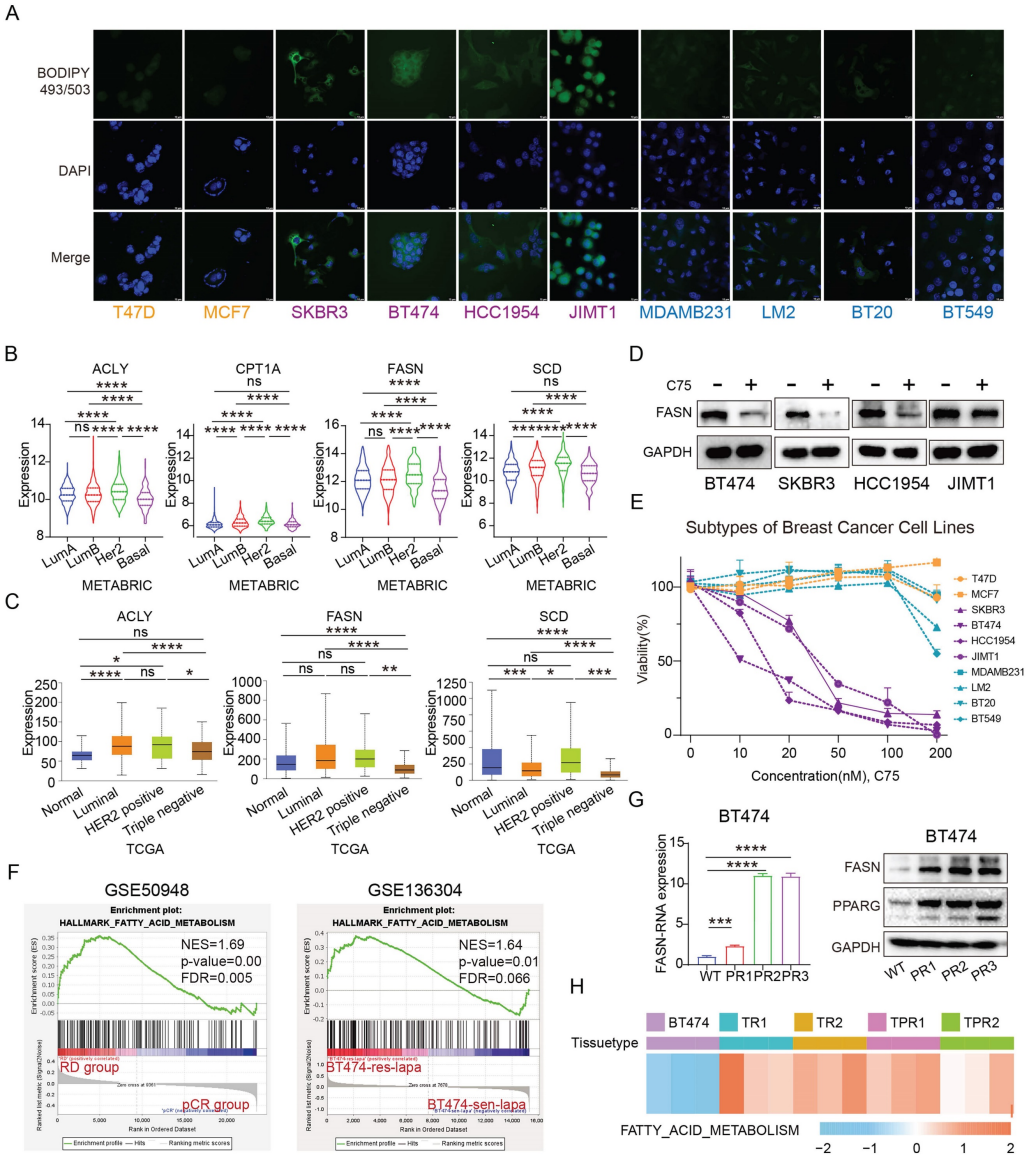
**Fatty acid metabolism is more active in anti-HER2 resistant HER2-positive breast cancer.** (A) Fatty acid distribution in different breast cancer cells (yellow for Luminal type, purple for HER2-positive, and blue for Triple-negative); (B) Expression of ACLY,CPT1A, FASN and SCD in different subtypes of breast cancer in METABRIC database; (C) Expression of ACLY, FASN and SCD in different subtypes of breast cancer in TCGA database; (D) Protein expression of FASN after treatment with C75 in BT474, SKBR3, HCC1954, JIMT1 cells; (E) Viability of different breast cancer cells treated with C75; (F) GSEA analysis of the fatty acid metabolism pathway in the GSE50948 dataset (RD vs pCR) and GSE136304 dataset (LR vs WT); (G) FASN expression in pyrotinib-resistant BT474 cells; (H) Enrichment of fatty acid metabolism pathways in single or dual-target resistant cell lines from the GSE121105 dataset. ACLY: ATP Citrate Lyase; CPT1A: Carnitine Palmitoyltransferase 1A; FASN: Fatty Acid Synthesis; SCD: Stearoyl-CoA Desaturase.

BODIPY 493/503 is a neutral fatty acid fluorescent probe that allows quantification of neutral fatty acid content via flow cytometry or visualization of fatty acid levels via confocal microscopy after staining^20^,^21^. Using BODIPY 493/503 stain, we observed that HER2-positive breast cancer cells exhibit higher fatty acid content compared to luminal and TNBC cells (**Figure 4A**). Analysis of the METABRIC database revealed that genes related to fatty acid metabolism, such as ACLY, CPT1A, FASN and SCD, are most highly expressed in HER2-positive breast cancer (**Figure 4B**). Consistent findings were also obtained from The Cancer Genome Atlas (TCGA) database data (**Figure 4C**). C75, a fatty acid synthase inhibitor^22^, significantly reduced FASN expression (**Figure 4D**). Treatment of breast cancer cells from different subtypes with C75 revealed that HER2-positive breast cancer cells were more sensitive to treatment with C75 (**Figure 4E**).

Sequencing data from patients treated with trastuzumab in the NOAH study showed significant activation of the fatty acid metabolic pathway in patients with residual cancer lesions compared to those with complete response, and analysis of the GSE136304 data revealed fatty acid metabolic activation in lapatinib-resistant BT474 cells compared to wild-type cells (**Figure 4F**). In addition, the expression of FASN was elevated in three pyrotinib-resistant cell lines (**Figure 4G**). Similarly, the GSE121105 data indicate an up-regulation of this pathway in the trastuzumab-resistant and trastuzumab/pertuzumab-resistant cell lines (**Figure 4H**). These findings suggest that fatty acid metabolism is more vigorous in HER2-positive breast cancer, and elevated fatty acid metabolic pathway is a consistent trait in HER2-positive breast cancer cells and tissues that are resistant to anti-HER2 treatment.

### PPARG activates fatty acid metabolic pathways in HER2-positive breast cancer

**Figure 5:**
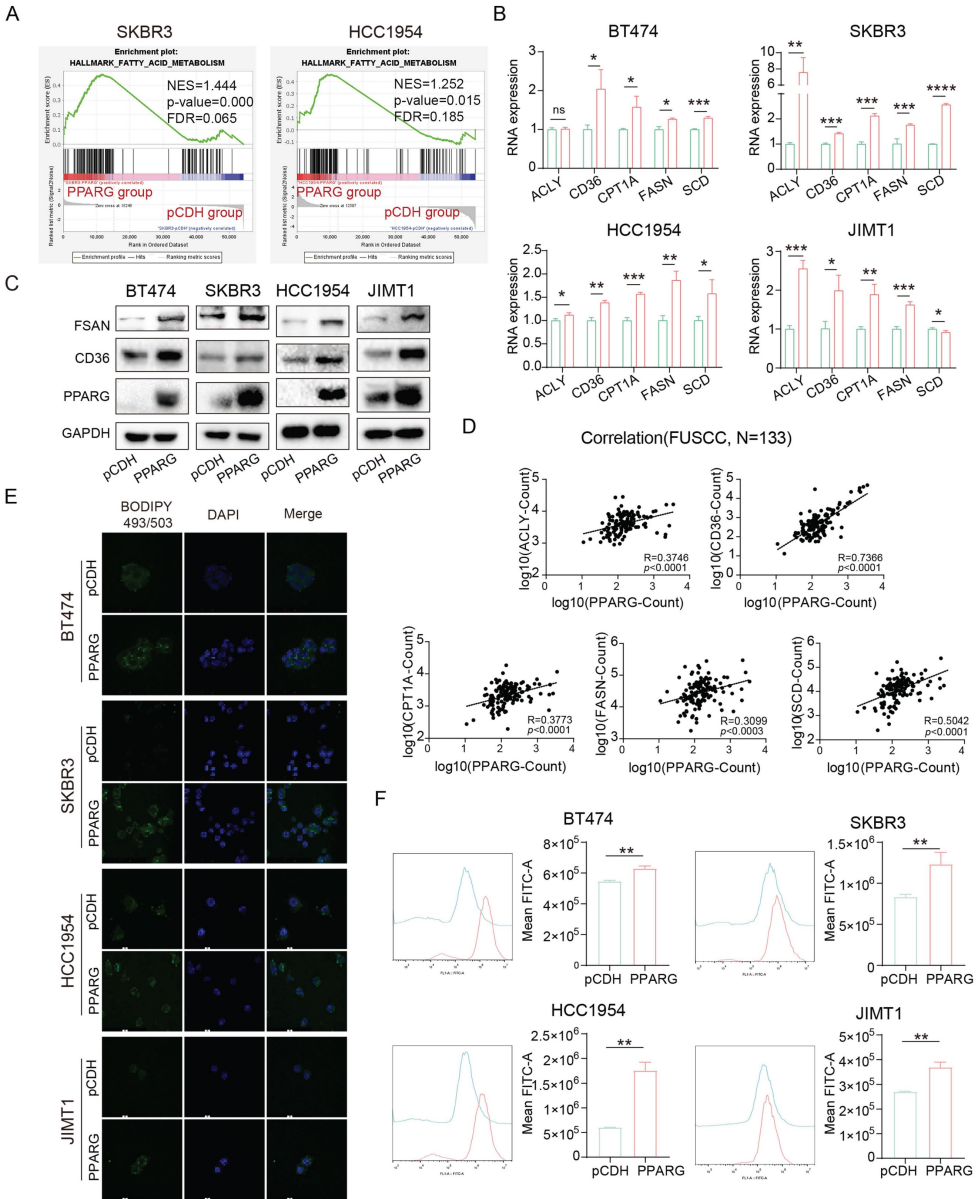
** PPARG activates fatty acid metabolic pathways in HER2-positive breast cancer.** (A) GSEA enrichment analysis of the fatty acid metabolism pathway with PPARG overexpressed and pCDH groups from SKBR3 and HCC1954 cell lines; (B) Expression levels of ACLY, CD36, CPT1A, FASN, and SCD in BT474, SKBR3, HCC1954, and JIMT1 PPARG-overexpressing cell lines; (C) Western blot of FASN and CD36 in PPARG overexpressing and pCDH group; (D) Correlation analysis of PPARG with ACLY, CD36, CPT1A, FASN, and SCD; (E) Fatty acid distribution in BT474, SKBR3, HCC1954, JIMT1 cells overexpressing PPARG compared to the pCDH groups; (F) Flow cytometry detection of lipid in BT474, SKBR3, HCC1954, JIMT1 cells overexpressing PPARG compared to the pCDH groups; CD36: Fatty Acid Translocase.

To investigate the impact of PPARG in HER2-positive breast cancer, we sequenced PPARG overexpressed and pCDH groups from SKBR3 and HCC1954 cell lines. Through GSEA enrichment analysis, we observed that the fatty acid metabolic pathway was activated in the PPARG overexpression group (**Figure 5A**). At the same time, we divided HER2-positive breast cancer patients in our center and METABRIC database into PPARG-low and PPARG-high expression groups based on median PPARG expression levels in tumor tissue. Through GSEA enrichment analysis, we also observed activation of fatty acid metabolic pathways in patients with high PPARG expression (**Supplementary figure 3A**).

At the RNA level, we found that PPARG promoted the expression of ACLY, CD36, CPT1A, FASN, and SCD (**Figure 5B**). Furthermore, at the protein level, the expression of FASN and CD36 was upregulated in PPARG overexpression cells and downregulated in PPARG gene knockout cells (**Figure 5C, Supplementary figure 3B**). In addition, in mice with tumor formation in situ, the tumor bodies of the group with high expression of PPARG also had higher expression levels of genes related to fatty acid metabolism, such as ACACA, ACLY and ACSS2 and so on (**Supplementary figure 3C**). In our sample of 133 patients, we explored the relationship between PPARG and essential fatty acid metabolism genes, uncovering a significant positive correlation between PPARG and ACLY, CD36, CPT1A, FASN, and SCD (**Figure 5D**). Additionally, according to the METABRIC database, PPARG demonstrated a notable positive correlation with CD36 and FASN (**Supplementary figure 3D**). Stained with BODIPY 493/503, the findings demonstrate an elevation of fatty acid content in HER2-positive breast cancer cells following PPARG overexpression (**Figure 5E, F**), whereas a decrease in fatty acid content is observed upon PPARG gene knockout (**Supplementary figure 3E, F**).

### PPARG enables the PI3K/Akt/mTOR signaling pathway by facilitating FASN

**Figure 6:**
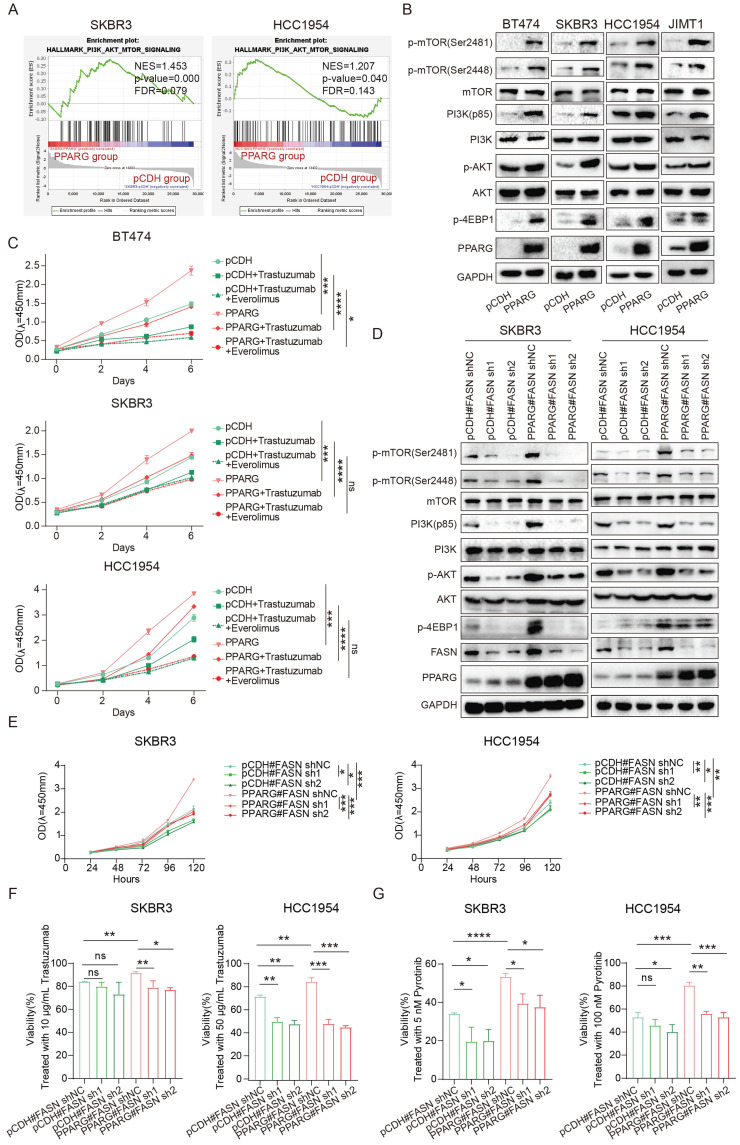
** PPARG enables the PI3K/Akt/mTOR signaling pathway by facilitating FASN.** (A) GSEA enrichment analysis of the PI3K/Akt/mTOR signaling with PPARG-overexpressed and pCDH groups from SKBR3 and HCC1954 cell lines; (B) Expression levels of PI3K/Akt/mTOR signaling pathway proteins in BT474, SKBR3 HCC1954 and JIMT1 cells PPARG-overexpressing group and pCDH group; (C) Proliferation curves of BT474, SKBR3 and HCC1954 cells PPARG-overexpressing group and pCDH group treated with or without Trastuzumab/ Everolimus; (D)Expression levels of PI3K/Akt/mTOR signaling pathway proteins in SKBR3 and HCC1954 knocking down FASN in PPARG overexpressing cells; (E) Proliferation curves of SKBR3 and HCC1954 knocking down FASN in PPARG overexpressing cells; (F) Viability of SKBR3 and HCC1954 cells treated with trastuzumab; (G) Viability of SKBR3 and HCC1954 cells treated with pyrotinib.

The GSEA analysis revealed a profound activation of the PI3K/Akt/mTOR (PAM) signaling pathway in PPARG overexpressed groups of SKBR3 and HCC1954 cell lines compared with pCDH group (**Figure 6A**). Based on data from our center and METABRIC databases, deep activation of the PAM signaling pathway was observed in individuals exhibiting elevated levels of PPARG expression (**Supplementary figure 4A**). The results showed that overexpression of PPARG significantly upregulated p-mTOR (Ser2481), p-mTOR (Ser2448), PI3K (p85), p-AKT and p-4EBP1 (**Figure 6B**). After knocking down PPARG, the expression of p-mTOR (Ser2481), p-mTOR (Ser2448), PI3K (p85), p-AKT and p-4EBP1 was downregulated (**Supplementary figure 4B**).

To test whether activation of the PAM signaling pathway is responsible for resistance in HER2-positive breast cancer induced by PPARG, we introduced Everolimus, an mTOR inhibitor widely used in studies of PAM signaling pathway inhibition^25^. Through the proliferation experiment, we found that the proliferative ability of PPARG-overexpressed cells remained stronger than that of the pCDH group cells treated with the same anti-HER2 drugs, but once we treated the cells with Everolimus in combination with the anti-HER2 drugs, we found that the drug resistance and proliferative effect caused by PPARG significantly weakened or even disappeared (**Figure 6C, Supplementary figure 4C, D**). This result demonstrates that the activation of the PAM signaling pathway is at the root of the reduced drug sensitivity promoted by PPARG in HER2-positive breast cancer.

The PAM signaling pathway is significantly activated in the pyrotinib-resistant cell lines (**Supplementary figure 4E**). Noteworthy in tumor cell biology, FASN orchestrates *de novo* fatty acid biosynthesis and underpins drug resistance in HER2-positive breast cancer^23^,^24^. FASN was less expressed in JIMT1 and more expressed in BT474, SKBR3, and HCC1954 in four HER2-positive breast cancer cell lines (**Supplementary figure 4F**). Combined with the expression of PPARG, we knocked down FASN in SKBR3 and HCC1954 PPARG-overexpressed cells*.* Pertinently, downregulation of FASN in PPARG-overexpressing cells precipitated a considerable reduction in p-mTOR (Ser2481), p-mTOR (Ser2448), PI3K (p85), p-AKT and p-4EBP1 levels (**Figure 6D**). *In vitro* investigations have demonstrated a decelerated cell proliferation in HER2-positive breast cancer cells following knockdown of FASN following PPARG overexpression (**Figure 6E**). In addition, after treating cells with a certain concentration of trastuzumab (**Figure 6F**), pyrotinib (**Figure 6G**) and lapatinib (**Supplementary figure 4G**), knocking down FASN in PPARG overexpressing cells significantly decreased cell survival, indicating an increased sensitivity of cells to HER2 targeted therapy drugs.

### GW9662 boosts drug sensitivity in HER2-positive breast cancer

**Figure 7:**
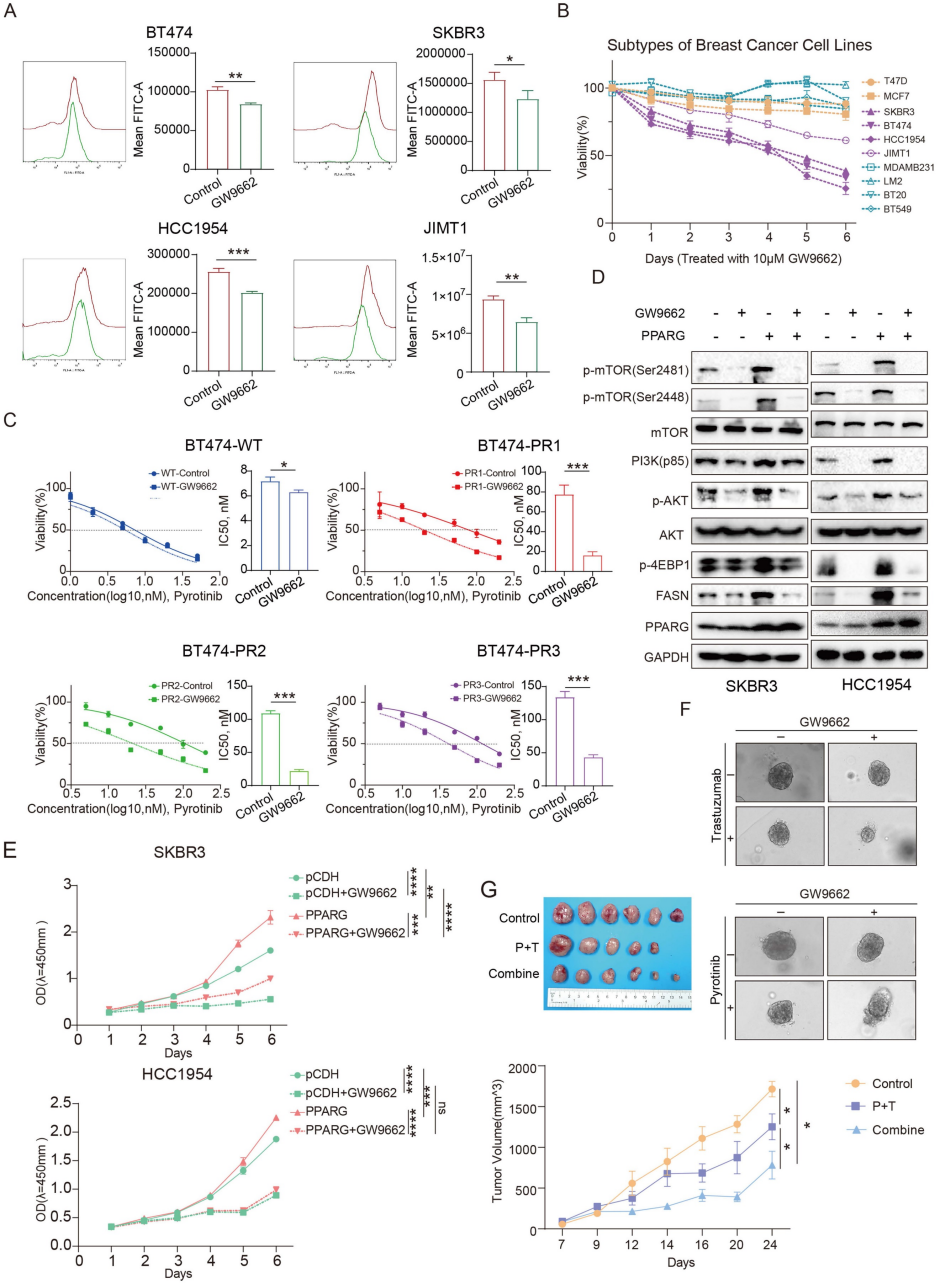
** ​GW9662 boosts drug sensitivity in HER2-positive breast cancer.** (A) Flow cytometry detection of lipid in BT474, SKBR3, HCC1954, JIMT1 cells treated with GW9662; (B) Proliferation curves of different breast cancer cells treated with 10 μM GW9662 (yellow for Luminal type, purple for HER2-positive, and blue for Triple-negative); (C) Gradient concentrations of pyrotinib treatment WT and PR BT474 cells treated with GW9662 were plotted for IC50 curves and IC50 value bar graphs; (D) Expression levels of PI3K/Akt/mTOR signaling pathway proteins in SKBR3 and HCC1954 PPARG-overexpressing cells with or without 10 μM GW9662; (E) Proliferation curves of SKBR3 and HCC1954 PPARG-overexpressing cells with or without 10 μM GW9662; (F) Morphological changes of PDOs with or without GW9662 or with or without trastuzumab/pyrotinib; (G) Schematic representation, and proliferation curves of in situ tumors in Control, P+T, and Combine groups.

GW9662, an inhibitor of PPARG^26^, it was able to inhibit key genes involved in fatty acid metabolism (**Supplementary figure 5A**), and it reduced the accumulation of fatty acids in HER2-positive breast cancer cells (**Figure 7A**). What's more, it shows a significant suppression of proliferation in HER2-positive breast cancer cell lines compared to Luminal and TNBC cell lines by treating with GW9662 (**Figure 7B**). When GW9662 is present, we found that the sensitivity of BT474 pyrotinib-resistant cell line to pyrotinib was significantly enhanced (**Figure 7C**), indicating that GW9662 can reverse the drug resistance of HER2-positive breast cancer cells and make them sensitive to the drug again. After GW9662 treated PPARG-overexpressed cells, the PAM signaling pathway could be inhibited (**Figure 7D**). GW9662 also inhibited the proliferation of PPARG- overexpressed cell lines (**Figure 7E**).

We combined GW9662 with trastuzumab, pyrotinib or lapatinib to treat HER2-positive breast cancer cells and organoids. Results demonstrated that GW9662 co-administration resulted in significantly greater cytotoxicity against compared to treatment with either trastuzumab, pyrotinib or lapatinib alone (**Figure 7F, Supplementary figure 5B, C, D**). In an *in vivo* study, in comparison to the control and paclitaxel/trastuzumab (P+T) groups, combined treatment with GW9662 significantly inhibited tumor growth in mice, resulting in a substantial reduction in tumor volume and mass (**Figure 7G, Supplementary figure 5E**), and flow cytometry results showed a significant decrease in fatty acid fluorescence values in the group treated with GW9662 in combination (**Supplementary figure 5F**). Oil red staining experiments also demonstrated a significant reduction in neutral fatty acids such as triglycerides in the group treated with GW9662 in combination (**Supplementary figure 5G**). The above results suggest that GW9662, a PPARG inhibitor, could be used as a sensitizing agent in anti-HER2 therapies with some clinical translational value.

### 13(S)-HODE promotes PPARG expression to promote drug resistance

**Figure 8:**
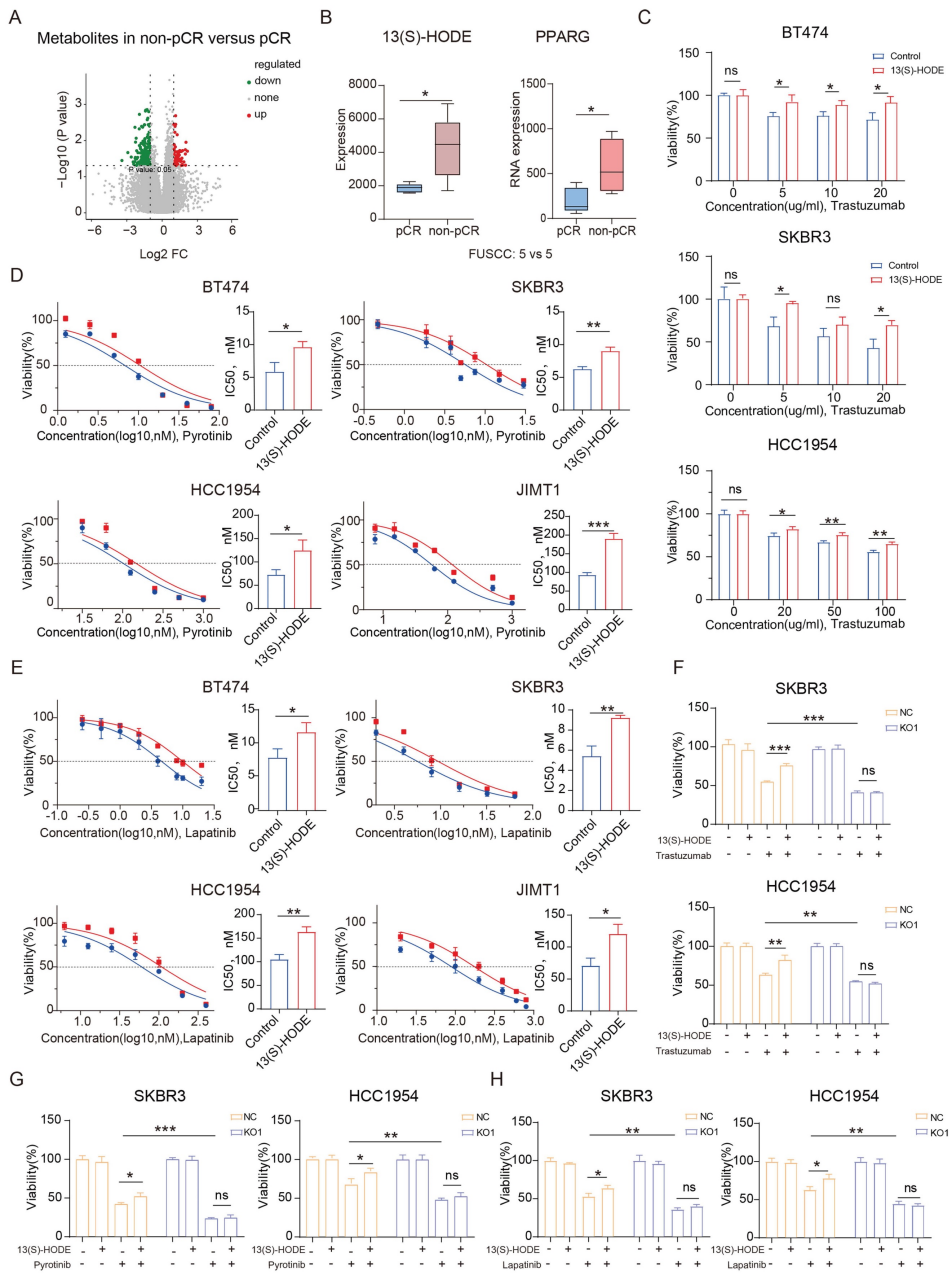
** 13(S)-HODE promotes PPARG expression to promote drug resistance.** (A) Volcano plot depicting differential metabolites between the non-pCR group and pCR groups; (B) Box plot illustrating the abundance of 13(S)-HODE and expression of PPARG in the pCR and non-pCR groups; (C) Viability rates of BT474, SKBR3 and HCC1954 cells after trastuzumab treatment with or without 13(S)-HODE; (D) IC_50_ curves and bar graph illustrating the gradient concentration of pyrotinib treatment in BT474, SKBR3, HCC1954 and JIMT1 cells when treated with or without 13(S)-HODE; (E) IC_50_ curves and bar graph illustrating the gradient concentration of lapatinib treatment in BT474, SKBR3, HCC1954 and JIMT1 cells when treated with or without 13(S)-HODE; (F) Viability of SKBR3 and HCC1954 NC and KO1 cells treated with or without 13(S)-HODE and trastuzumab; (G) Viability of SKBR3 and HCC1954 NC and KO1 cells treated with or without 13(S)-HODE and pyrotinib; (H) Viability of SKBR3 and HCC1954 NC and KO1 cells treated with or without 13(S)-HODE and lapatinib.

We collected core needle biopsy (CNB) specimens from 10 HER2-positive breast cancer patients who achieved and did not achieve pCR after standard neoadjuvant therapy. Using untargeted metabolomics to measure the abundance of various metabolites in the samples, we identified a total of 241 differential metabolites, with 57 metabolites showing high abundance (fold change ≥2) in the non-pCR group and 184 metabolites showing low abundance (fold change ≤1/2) (**Figure 8A**). KEGG enrichment analysis of the differential metabolites revealed significant activation of the PPAR signaling pathway in the non-pCR group (**Supplementary figure 6A**), characterized by higher abundance of 13-(S)-hydroperoxylinolenic acid (13(S)-HODE, C_18_H_30_O_4_) in the non-pCR group (**Figure 8B**). Moreover, PPARG expression was higher in the non-pCR group.

HER2-positive breast cancer cells cultured with stable 13(S)-HODE concentration exhibited increased PPARG expression (**Supplementary figure 6B**). In a consistent trastuzumab, pyrotinib and lapatinib concentration setting, cell survival rates were higher in the presence of 13(S)-HODE (**Figure 8C, D, E**). Colony formation assays indicated a higher number of clones in HER2-positive breast cancer cells treated with both trastuzumab and 13(S)-HODE (**Supplementary figure 6C**), the results were also consistent with the treatment with pyrotinib (**Supplementary figure 6D**) and lapatinib (**Supplementary figure 6E**). Knockout of PPARG reduced the impact of 13(S)-HODE on anti-HER2 drug sensitivity, particularly for trastuzumab, pyrotinib and lapatinib (**Figure 8F, G, H**). The above results demonstrate that 13(S)-HODE can reduce the sensitivity of HER2-positive breast cancer to anti-HER2 therapy by targeting PPARG.

## Discussion

Metabolic reprogramming serves as a key hallmark of cancer^27^, providing auspicious circumstances for continued growth, differentiation, etc^28^. Among various metabolic alterations, the crucial role of fatty acid metabolism in cancer has received considerable scrutiny^29^. Fatty acids actively participate in membrane synthesis during the rapid proliferation of cancer cells and function as vital energy reservoirs during periods of stress or altered cellular conditions^30^,^31^. The transport, synthesis, and breakdown of fatty acids have garnered significant attention as potential therapeutic targets in cancer, with key enzymes and genes in this pathway being the focus of extensive research^23^,^32^. Previous studies have identified high expression of fatty acid metabolism-related genes such as PLIN1, CPT1A, FABP4, ACOX1, and FASN in HER2-positive breast cancer^33^. Our research also confirms that HER2-positive breast cancer exhibits more active fatty acid metabolism compared to other subtypes and shows increased sensitivity to fatty acid synthesis inhibitors. It also underscores the importance of exploring the metabolic profile of cancer subtypes, which insights may contribute to the development of more personalized and effective treatment regimens for HER2-positive breast cancer patients.

We also observed elevated fatty acid synthase activity and enhanced fatty acid metabolism in anti-HER2 resistant cell lines. In the context of HER2-positive breast cancer, genes including ACLY, FASN, CPT1/2, CD36, and SCD1 have been associated with drug sensitivity to targeted therapies 34,35. Among these, investigations into the role of FASN in modulating drug sensitivity in HER2-positive breast cancer have been particularly profound.

Elevated HER2 expression activates FASN activity, fostering cancer cell proliferation, while FASN amplifies the HER2-mediated signaling pathway^36^. In HER2-positive breast cancer patients who have developed resistance toanti-HER2 therapies like trastuzumab or lapatinib, FASN inhibitors have shown promise in effectively reversing resistance, potentially serving as a sensitization strategy for HER2-targeted therapies^35^. For instance, the FASN inhibitor TVB-2640 has demonstrated potential in the treatment of advanced HER2-positive breast cancer when combined with chemotherapy and trastuzumab^37^. Additionally, the up-regulation of the fatty acid transporter protein CD36 in lapatinib-resistant BT474 cells leads to increased exogenous fatty acid uptake and enhanced metabolic plasticity in the resistant cell line^38^. In the NeoALTTO clinical trial, high expression of CD36 was associated with a poor response to neoadjuvant therapy and an unfavorable prognosis in HER2-positive breast cancer patients^39^. This research has unveiled the promoting role of PPARG in the fatty acid metabolism pathway, thereby fostering the enhancement of intracellular fatty acid levels through the up-regulation of critical enzymes such as FASN and CD36. We therefore propose that activation of the fatty acid metabolism pathway is the underlying mechanism by which PPARG initiates resistance in HER2-positive breast cancer.

The PI3K/Akt/mTOR signaling pathway is primarily triggered by the activation of receptor tyrosine kinases (RTKs), which enhance the activity of the PI3K complex. This leads to the conversion of phosphatidylinositol-4, 5-bisphosphate to phosphatidylinositol-3, 4, 5-triphosphate (PIP3). PIP3 then undergoes phosphorylation to activate AKT, which in turn activates the mTOR complex through a series of phosphorylation events^41^,^42^. It serves as a pivotal axis in tumorigenesis, orchestrating an array of cellular processes including proliferation, differentiation, apoptosis, angiogenesis, drug response, and metastasis in breast cancer^43^. HER-2 forms heterodimers that subsequently activate downstream signaling pathways, including the PAM signaling pathway. Aberrant activation of the PAM signaling pathway is closely associated with resistance to anti-HER-2 therapy^44^. Studies reveal that patients with HER2-positive breast cancer harboring concurrent mutations in PIK3CA and PTEN exhibit a proclivity towards trastuzumab resistance^45^. Notably, a combinatorial approach employing PAM inhibitors demonstrates efficacy in enhancing progression-free survival within this patient cohort^46^., suggesting a pivotal role for this pathway in mediating resistance. Moreover, the PI3K/Akt pathway exerts regulatory control over fatty acid biosynthesis via post-transcriptional mechanisms, orchestrating the activation of the SREBP transcription factor and subsequent synthesis of fatty acids and steroids^47^. Conversely, activation of the fatty acid metabolism pathway positively modulates the PAM signaling cascade^48^. Indeed, targeted inhibition of FASN has been shown to efficiently attenuate mTOR phosphorylation and downstream signaling molecules^49^. Our research elucidates the role of PPARG in augmenting the PAM signaling cascade, mTOR inhibitors can restore sensitivity to anti-HER2 drugs in PPARG overexpressing cells. While PAM signaling pathway is deactivated by FASN knockdown, and demonstrating that FASN inhibition effectively counteracts PPARG-induced pro-resistance and pro-proliferative tendencies. The reversible effect of FASN knockdown on PAM signaling pathway highlights its potential as a target for therapeutic intervention.

Multiple large-scale real-world clinical studies have underscored the significant impact of nutritional status on the onset and prognosis of breast cancer^50^. Specifically, in early-stage HR-negative/HER2-positive breast cancer, obese patients demonstrate a heightened risk of recurrence compared to those with a normal BMI (HR=2.29, 95% CI: 1.01-5.20, p=0.047) 51. Moreover, among HER2-positive breast cancer patients who have undergone neoadjuvant therapy (NAT), individuals classified as obese exhibit a diminished pathological complete response (pCR) rate in contrast to those with normal weight^52^,^53^. Consequently, obesity emerges as a notable risk factor for attenuated NAT efficacy in HER2-positive breast cancer patients^54^. In addition to genetic predispositions, environmental factors such as dietary patterns play a pivotal role in shaping human nutritional status, with unfavorable dietary compositions posing various disadvantages in cancer onset and progression^55^. 13(S)-HODE, a constituent of the linoleic acid family of essential fatty acids, has been the subject of previous investigations revealing a positive correlation between dietary linoleic acid and α-linolenic acid levels and BMI^56^. This suggests that a linoleic acid-rich diet may contribute to elevated levels of 13(S)-HODE in breast cancer tissue, consequently diminishing the efficacy of neoadjuvant therapy in affected patients. Notably, this study elucidates the role of 13(S)-HODE in attenuating the sensitivity of HER2-positive breast cancer to anti-HER2 therapy. Regrettably, the limited sample size of merely 10 subjects precluded a comprehensive validation of the correlation between the linoleic acid diet and 13 (S)-HODE. Building upon our discoveries, it is prudent to postulate that t an increase in the 13(S)-HODE content in a linoleic acid-rich diet, may lead to poor anti-HER2 treatment outcomes in HER2-positive breast cancer patients, and this effect is mediated through its promotion of PPARG activity, contributing to resistance.

Notably, GW9662, a specific PPARG inhibitor, has demonstrated efficacy in slowing the progression of non-alcoholic fatty liver disease in humans^57^. GW9662 exhibits the ability to suppress stem cell-related genes (KLF4 and ALDH), leading to apoptosis induction and inhibition of tumor sphere formation^17^. Nur77 has been identified as an inhibitor of breast cancer development. However, PPARG interacts with Nur77, facilitating its degradation via ubiquitin ligase trim13-mediated ubiquitination, thereby counteracting Nur77's inhibitory effect on HER2-positive breast cancer^18^. As a result, PPARG emerges as a pivotal pro-carcinogenic factor in HER2-positive breast cancer, correlating with a poorer long-term prognosis and serving as a clinically significant biomarker. Additionally, PPARG dampens the responsiveness of HER2-positive breast cancer to anti-HER2 drugs. PPARG inhibitor GW9662 can sensitize pyrotinib-resistant cell lines to pyrotinb again. After treatment with GW9662, the activation degree of fatty acid metabolism and PAM signaling pathway can be reduced, and the malignant proliferation rate of cells can be slowed down. In both *in vitro* and *in vivo* experiments, combining anti-HER2 agents with GW9662 resulted in significantly amplified inhibition of cell proliferation or tumor growth compared to single agent administration. This underscores the promising utility of GW9662 in the therapeutic arsenal against HER2-positive breast cancer as a sensitizing strategy for anti-HER2 therapies.

## Conclusion

**Figure 9:**
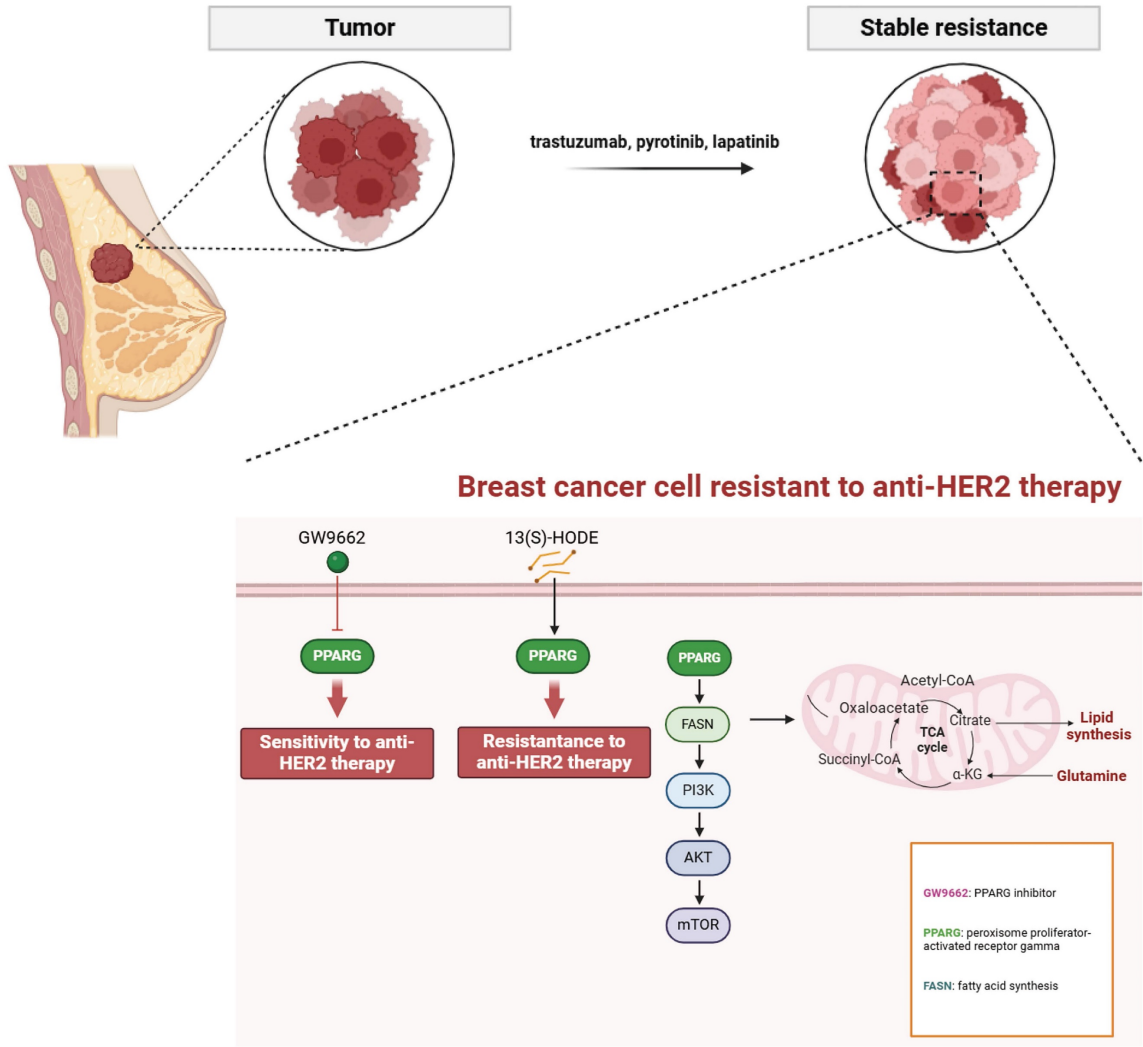
Mechanistic diagram of PPARG-mediated fatty acid metabolism and PI3K/Akt/mTOR signaling pathway in promoting drug resistance in HER2-positive breast cancer.

PPARG plays a crucial role in determining the efficacy and prognosis of HER2-positive breast cancer. HER2-positive breast cancer resistant cells and tissues showed activation of fatty acid metabolic pathway and PI3K/AKT/mTOR signaling pathway. Through its involvement in boosting fatty acid metabolism, PPARG finely tunes the activation of the PI3K/Akt/mTOR pathway, affecting both drug sensitivity and cancer cell growth. Excitingly, the PPARG inhibitor GW9662 holds great potential for practical use in anti-HER2 therapy (**Figure 9**).

## Materials and Methods

### Patients and ethical statement

Tissue samples from HER2-positive breast cancer patients prior to neoadjuvant therapy were collected from the Department of Breast Surgery at Fudan University Shanghai Cancer Center (FUSCC), Shanghai, China. These samples were preserved in RNA later and stored at -80°C for long-term use. RNA-Seq data from 133 HER2-positive breast cancer patients were generated by FUSCC, and tissue samples from 193 HER2-positive breast cancer patients who underwent surgical resection at FUSCC in 2012 were collected with subsequent follow-up. All patients provided informed consent for the use of their resected tissues in research.

GSE121105, GSE136304, GSE52707, GSE50948 and GSE114082 dataset from GEO database (https://www.ncbi.nlm.nih.gov/geo/), TCGA database (https://cancergenome.nih.gov/) and METABRIC database (https://www.bccrc.ca/dept/mo/) patient sequencing and follow-up data were obtained through official channels.

### GEO public dataset and ROC analysis

**GEO Dataset Analysis:** To analyze public datasets, visit the GEO database and enter the chip series number (Series ID) in the search box. On the results page, click "Analyze with GEO2R" to access the GEO2R analysis tool. In the "Samples" table, define groups and initiate differential analysis by clicking "Analyze" using default parameters. The output includes a list of significantly differentially expressed genes.

**ROC Analysis:** For receiver operating characteristic (ROC) curve analysis, access the ROC Plotter website and select "ROC Plotter for Breast Cancer." Enter the target gene in the search box and specify the treatment as "Anti-HER2 therapy Any" or "Trastuzumab" under the "Treatment" field. In the "HER2 status" field, select "Positive" to refine the analysis. Click "Calculate" to generate an ROC curve, which illustrates the association between the target gene and the efficacy of anti-HER2 therapy or trastuzumab treatment.

### Cell lines, culture conditions, and reagents

BT474, SKBR3, HCC1954, JIMT1, T47D, MCF7, MDAMB231, LM2, BT20, BT549, and 293T cell lines were confirmed mycoplasma-free (Vazyme) and maintained in media supplemented with 10% fetal bovine serum (FBS, Gibco), 100 IU/mL penicillin, and 100 µg/mL streptomycin (Invitrogen) at 37°C in a humidified incubator with 5% CO₂.

GW9662 and 13(S)-HODE were purchased from GlpBio. Trastuzumab, pyrotinib, and lapatinib were obtained from FUSCC. Antibodies against GAPDH, FASN, and CD36 were sourced from Proteintech, while those targeting PPARG, mTOR, p-mTOR (Ser2448), p-mTOR (Ser2481), PI3K (p85), PI3K, p-AKT, AKT, and p-4EBP1 were acquired from Cell Signaling Technology.

### Primers and sequences

### Establishment of pyrotinib-resistant BT474 cell lines

BT474 cells were cultured in RPMI 1640 medium supplemented with pyrotinib at increasing concentrations (0, 50, 100, 200 nM). Once cell density decreased to 30%-40%, the medium was replaced with pyrotinib-free medium. This process was repeated until cells exhibited stable growth in pyrotinib-containing medium. The IC_50_ value of resistant cells was subsequently determined.

### IC50 measurement

Cells were seeded in 96-well plates at a density of 4000-8000 cells per well in 100 μL of culture medium, with three replicate wells per group. After 24 hours, a gradient of drug concentrations was prepared and added. Cell viability was assessed after 3-7 days using the CCK-8 assay, and absorbance at 450 nm was measured to determine the IC_50_ value. The cell survival rate was calculated as: Survival rate=100% × [(Experimental group - Blank group)/ (Control group - Blank group)].

### Plate colony formation assay

**(A) Cell Proliferation Assay**: Cells were seeded at 1000-3000 cells per well in 6-well plates with 2 mL of culture medium. The medium was replaced every 2-3 days. Once visible colonies formed, cells were stained with crystal violet for imaging.

**(B) Drug Sensitivity Assay:** Cells were seeded at 20000-100000 cells per well in 6-well plates and allowed to adhere for 24 hours. Culture medium containing the designated drug concentration was added, and cells were continuously exposed for a defined period. Once visible colonies formed, cells were stained with crystal violet for imaging.

### BODIPY 493/503 staining and flow cytometry

A 2 μM BODIPY 493/503 staining solution was prepared in PBS. Cells were incubated with the staining solution at 37°C for 15 minutes in the dark. After incubation, cells were rinsed with 3 mL of PBS and trypsinized to obtain a single-cell suspension. Cells were resuspended in 5 mL of PBS, centrifuged at 250g for 5 minutes at 4°C, and washed again with 3 mL of PBS before a final centrifugation. The cell pellet was resuspended in 300 μL of 1× flow cytometry buffer, and fluorescence intensity was measured using flow cytometry.

### Oil red O staining in tissue samples

Frozen tissue sections stored at -20°C were thawed for 5-10 minutes. Stain remover solution was applied for 20 seconds, followed by staining with a working Oil Red O solution for 10-20 minutes. After staining, sections were washed with an appropriate stain wash solution, immersed in ddH₂O for 20 seconds, and imaged under a microscope, either directly or after sealing with a coverslip.

### Metabolite extraction from human breast cancer CNB tissue

CNB samples were retrieved from liquid nitrogen, cut into small pieces, and weighed (100 mg per sample). The tissue was ground in liquid nitrogen, and 120 μL of a 1:1 methanol/ddH₂O mixture was added. The mixture was incubated at room temperature for 10 minutes, then stored at -20°C for 12 hours. Following centrifugation at 4,000g for 20 minutes, the supernatant was transferred to a 96-well plate. A 10 μL aliquot from each sample was taken for standardization, and the remaining samples were stored at -80°C for further mass spectrometry analysis.

### In vivo tumor formation assay

Female BALB/c nude mice (5 weeks old) were obtained from the Animal Experimental Center of FUSCC. Tumor volume was calculated using the formula: V = 1/2 (long diameter × short diameter²), Tumor volume was maintained below 2000 mm³.

**(A) PPARG promotes the proliferation of HER2-positive breast cancer cells**: Twelve mice were randomly divided into two groups. JIMT1 pCDH and JIMT1 PPARG cells were harvested, digested, and prepared as a suspension containing 1.5 × 10⁶ cells in 100 µL. The suspension was mixed with Matrigel and PBS at a 1:1 ratio before implantation. Tumor formation was monitored, and tumor size was measured every 2-3 days. Body weight was recorded.

**(B) GW9662 in combination with anti-HER2 therapy against HER2-positive breast cancer**: Eighteen mice were randomly assigned to three groups. JIMT1 wild-type cells were harvested, digested, and suspended in 100 µL of PBS (1.5 × 10⁶ cells), mixed with Matrigel and PBS at a 1:1 ratio. Once tumors reached an average volume of 100 mm³, treatment was initiated. The groups included: ① **Control group:** No treatment, ② **P+H group:** Treated with trastuzumab (10 mg/kg) and paclitaxel (20 mg/kg) via intraperitoneal injection, ③ **Combination group:** Treated with trastuzumab (10 mg/kg), paclitaxel (20 mg/kg), and GW9662 (10 mg/kg) via intraperitoneal injection. Drug administration occurred every three days, with tumor size and body weight recorded.

### Western blot analysis

Cells were lysed in RIPA buffer supplemented with 1% protease and phosphatase inhibitors. Lysis was performed using a sonicator for 20 seconds, repeated five times with 10-second intervals. Protein concentration was measured using a BCA protein assay kit (Thermo Fisher). Samples were separated on 10% SDS-PAGE gels and transferred to PVDF membranes. Membranes were blocked with 5% non-fat milk in TBST for 1 hour, followed by overnight incubation with primary antibodies at 4°C. After washing, membranes were incubated with HRP-conjugated secondary antibodies, and proteins were visualized using an ECL Western Blotting Substrate.

### Statistical analysis

Data were presented as mean ± standard deviation (SD) from at least three independent experiments. Differences between two groups were assessed using an unpaired Student's t-test in Prism 8.0 (GraphPad). Kaplan-Meier survival curves were generated and compared using the log-rank test. For multiple-group comparisons, statistical significance was defined as: p < 0.05 (*), p < 0.01 (**), p < 0.001 (***), and p < 0.0001 (****).

## Supplementary Material

Supplementary figures and tables.

## Figures and Tables

**Table 1:** Multivariate Cox regression analysis of OS in HER2-positive breast cancer

Patient Characteristics		*p values*	HR (95%)
pN			
	pN0		
	pN1-3	**0.023**	2.622(1.139-6.034)
	Unknown		
PPARG expression			
	Low		
	High	**0.022**	2.484(1.117-5.304)

**Table 2:** Multivariate Cox regression analysis of DFS in HER2-positive breast cancer

Patient Characteristics		*p values*	HR (95%)
pN			
	pN0		
	pN1-3	**0.003**	2.605(1.384-4.901)
	Unknown		
PPARG expression			
	Low		
	High	**0.026**	1.948(1.083-3.505)

**Table 3:** Primers and sequences

Primers for CRISPR		
crspPPARG1-S		CACCGCAACTTTGGGATCAGCTCCG
crspPPARG1-AS		AAACCGGAGCTGATCCCAAAGTTGC
crspPPARG2-S		CACCGATTCAGCTGGTCGATATCAC
crspPPARG2-AS		AAACGTGATATCGACCAGCTGAATC
crspPPARG3-S		CACCGTATGAGACATCCCCACTGCA
crspPPARG3-AS		AAACTGCAGTGGGGATGTCTCATAC
Primers for PCR		
PPARG-S	ATTCTAGAGCTAGCGAATTCATGGTTGACACAGAGATGCCATTC	
PPARG-AS	ATGGTCTTTGTAGTCGGATCCGTACAAGTCCTTGTAGATCTCCTG	
Primers for shRNA		
FASN-sh1-oligo1		CCGGCCTACTGGATGCGTTCTTCAACTCGAGTTGAAGAACGCATCCAGTAGGTTTTTG
FASN-sh1-oligo2		AATTCAAAAACCTACTGGATGCGTTCTTCAACTCGAGTTGAAGAACGCATCCAGTAGG
FASN-sh2-oligo1		CCGGCATGGAGCGTATCTGTGAGAACTCGAGTTCTCACAGATACGCTCCATGTTTTTG
FSAN-sh2-oligo2		AATTCAAAAACATGGAGCGTATCTGTGAGAACTCGAGTTCTCACAGATACGCTCCATG
		

## References

[1] Siegel RL, Giaquinto AN, Jemal A (2024). Cancer statistics, 2024. CA Cancer J Clin.

[2] Kreutzfeldt J, Rozeboom B, Dey N, De P (2020). The trastuzumab era: current and upcoming targeted HER2+ breast cancer therapies. Am J Cancer Res.

[3] Pernas S, Tolaney SM (2019). HER2-positive breast cancer: new therapeutic frontiers and overcoming resistance. Ther Adv Med Oncol.

[4] Harbeck N, Gnant M (2017). Breast cancer. Lancet.

[5] Yang M, Li Y, Kong L (2023). Inhibition of DPAGT1 suppresses HER2 shedding and trastuzumab resistance in human breast cancer. J Clin Invest.

[6] van Ramshorst MS, van der Voort A, van Werkhoven ED (2018). Neoadjuvant chemotherapy with or without anthracyclines in the presence of dual HER2 blockade for HER2-positive breast cancer (TRAIN-2): a multicentre, open-label, randomised, phase 3 trial. Lancet Oncol.

[7] Gianni L, Pienkowski T, Im YH (2016). 5-year analysis of neoadjuvant pertuzumab and trastuzumab in patients with locally advanced, inflammatory, or early-stage HER2-positive breast cancer (NeoSphere): a multicentre, open-label, phase 2 randomised trial. Lancet Oncol.

[8] Hurvitz SA, Martin M, Symmans WF (2018). Neoadjuvant trastuzumab, pertuzumab, and chemotherapy versus trastuzumab emtansine plus pertuzumab in patients with HER2-positive breast cancer (KRISTINE): a randomised, open-label, multicentre, phase 3 trial. Lancet Oncol.

[9] Zhu K, Yang X, Tai H, Zhong X, Luo T, Zheng H (2024). HER2-targeted therapies in cancer: a systematic review. Biomark Res.

[10] Amaral MVS, AJ DESP, EL DAS (2019). Establishment of Drug-resistant Cell Lines as a Model in Experimental Oncology: A Review. Anticancer Res.

[11] McDermott M, Eustace AJ, Busschots S (2014). In vitro Development of Chemotherapy and Targeted Therapy Drug-Resistant Cancer Cell Lines: A Practical Guide with Case Studies. Front Oncol.

[12] Xavier CP, Pesic M, Vasconcelos MH (2016). Understanding Cancer Drug Resistance by Developing and Studying Resistant Cell Line Models. Curr Cancer Drug Targets.

[13] Punyamurtula U, Brown TW, Zhang S, George A, El-Deiry WS (2023). Cancer cell seeding density as a mechanism of chemotherapy resistance: a novel cancer cell density index based on IC50-Seeding Density Slope (ISDS) to assess chemosensitivity. Am J Cancer Res.

[14] Chen X, Gu J, Huang J (2023). Characterization of circRNAs in established osimertinib-resistant non-small cell lung cancer cell lines. Int J Mol Med.

[15] Marion-Letellier R, Savoye G, Ghosh S (2016). Fatty acids, eicosanoids and PPAR gamma. Eur J Pharmacol.

[16] Liu L, Si N, Ma Y (2018). Hydroxysafflor-Yellow A Induces Human Gastric Carcinoma BGC-823 Cell Apoptosis by Activating Peroxisome Proliferator-Activated Receptor Gamma (PPARγ). Med Sci Monit.

[17] Wang X, Sun Y, Wong J, Conklin DS (2013). PPARγ maintains ERBB2-positive breast cancer stem cells. Oncogene.

[18] Yang PB, Hou PP, Liu FY (2020). Blocking PPARγ interaction facilitates Nur77 interdiction of fatty acid uptake and suppresses breast cancer progression. Proc Natl Acad Sci U S A.

[19] Tanner M, Kapanen AI, Junttila T (2004). Characterization of a novel cell line established from a patient with Herceptin-resistant breast cancer. Mol Cancer Ther.

[20] Qiu B, Simon MC (2016). BODIPY 493/503 Staining of Neutral Lipid Droplets for Microscopy and Quantification by Flow Cytometry. Bio Protoc.

[21] Lee W, Kim HY, Choi YJ (2022). SNX10-mediated degradation of LAMP2A by NSAIDs inhibits chaperone-mediated autophagy and induces hepatic lipid accumulation. Theranostics.

[22] Jin Q, Yuan LX, Boulbes D (2010). Fatty acid synthase phosphorylation: a novel therapeutic target in HER2-overexpressing breast cancer cells. Breast Cancer Res.

[23] Ferraro GB, Ali A, Luengo A (2021). FATTY ACID SYNTHESIS IS REQUIRED FOR BREAST CANCER BRAIN METASTASIS. Nat Cancer.

[24] Corominas-Faja B, Vellon L, Cuyàs E (2017). Clinical and therapeutic relevance of the metabolic oncogene fatty acid synthase in HER2+ breast cancer. Histol Histopathol.

[25] Casadevall D, Hernández-Prat A, García-Alonso S (2022). mTOR Inhibition and T-DM1 in HER2-Positive Breast Cancer. Mol Cancer Res.

[26] Baumann A, Burger K, Brandt A (2022). GW9662, a peroxisome proliferator-activated receptor gamma antagonist, attenuates the development of non-alcoholic fatty liver disease. Metabolism.

[27] Koundouros N, Poulogiannis G (2020). Reprogramming of fatty acid metabolism in cancer. Br J Cancer.

[28] Beloribi-Djefaflia S, Vasseur S, Guillaumond F (2016). Lipid metabolic reprogramming in cancer cells. Oncogenesis.

[29] Kuo CY, Ann DK (2018). When fats commit crimes: fatty acid metabolism, cancer stemness and therapeutic resistance. Cancer Commun (Lond).

[30] Tan SK, Hougen HY, Merchan JR, Gonzalgo ML, Welford SM (2023). Fatty acid metabolism reprogramming in ccRCC: mechanisms and potential targets. Nat Rev Urol.

[31] Snaebjornsson MT, Janaki-Raman S, Schulze A (2020). Greasing the Wheels of the Cancer Machine: The Role of Lipid Metabolism in Cancer. Cell Metab.

[32] Röhrig F, Schulze A (2016). The multifaceted roles of fatty acid synthesis in cancer. Nat Rev Cancer.

[33] Jung YY, Kim HM, Koo JS (2015). Expression of Lipid Metabolism-Related Proteins in Metastatic Breast Cancer. PLoS One.

[34] Azam A, Sounni NE (2022). Lipid Metabolism Heterogeneity and Crosstalk with Mitochondria Functions Drive Breast Cancer Progression and Drug Resistance. Cancers (Basel).

[35] Hoy AJ, Nagarajan SR, Butler LM (2021). Tumour fatty acid metabolism in the context of therapy resistance and obesity. Nat Rev Cancer.

[36] Vazquez-Martin A, Colomer R, Brunet J, Menendez JA (2007). Pharmacological blockade of fatty acid synthase (FASN) reverses acquired autoresistance to trastuzumab (Herceptin by transcriptionally inhibiting 'HER2 super-expression' occurring in high-dose trastuzumab-conditioned SKBR3/Tzb100 breast cancer cells. Int J Oncol.

[37] Falchook G, Infante J, Arkenau HT (2021). First-in-human study of the safety, pharmacokinetics, and pharmacodynamics of first-in-class fatty acid synthase inhibitor TVB-2640 alone and with a taxane in advanced tumors. EClinicalMedicine.

[38] Feng WW, Wilkins O, Bang S (2019). CD36-Mediated Metabolic Rewiring of Breast Cancer Cells Promotes Resistance to HER2-Targeted Therapies. Cell Rep.

[39] Ligorio F, Di Cosimo S, Verderio P (2022). Predictive Role of CD36 Expression in HER2-Positive Breast Cancer Patients Receiving Neoadjuvant Trastuzumab. J Natl Cancer Inst.

[40] Vanauberg D, Schulz C, Lefebvre T (2023). Involvement of the pro-oncogenic enzyme fatty acid synthase in the hallmarks of cancer: a promising target in anti-cancer therapies. Oncogenesis.

[41] Peng Y, Wang Y, Zhou C, Mei W, Zeng C (2022). PI3K/Akt/mTOR Pathway and Its Role in Cancer Therapeutics: Are We Making Headway?. Front Oncol.

[42] Pan L, Li J, Xu Q (2024). HER2/PI3K/AKT pathway in HER2-positive breast cancer: A review. Medicine (Baltimore).

[43] Fuso P, Muratore M, D'Angelo T (2022). PI3K Inhibitors in Advanced Breast Cancer: The Past, The Present, New Challenges and Future Perspectives. Cancers (Basel).

[44] Vitale SR, Martorana F, Stella S (2021). PI3K inhibition in breast cancer: Identifying and overcoming different flavors of resistance. Crit Rev Oncol Hematol.

[45] Fujimoto Y, Morita TY, Ohashi A (2020). Combination treatment with a PI3K/Akt/mTOR pathway inhibitor overcomes resistance to anti-HER2 therapy in PIK3CA-mutant HER2-positive breast cancer cells. Sci Rep.

[46] André F, O'Regan R, Ozguroglu M (2014). Everolimus for women with trastuzumab-resistant, HER2-positive, advanced breast cancer (BOLERO-3): a randomised, double-blind, placebo-controlled phase 3 trial. Lancet Oncol.

[47] Horton JD, Goldstein JL, Brown MS (2002). SREBPs: activators of the complete program of cholesterol and fatty acid synthesis in the liver. J Clin Invest.

[48] Ventura R, Mordec K, Waszczuk J (2015). Inhibition of de novo Palmitate Synthesis by Fatty Acid Synthase Induces Apoptosis in Tumor Cells by Remodeling Cell Membranes, Inhibiting Signaling Pathways, and Reprogramming Gene Expression. EBioMedicine.

[49] Han L, Dai W, Luo W (2023). Enhanced De Novo Lipid Synthesis Mediated by FASN Induces Chemoresistance in Colorectal Cancer. Cancers (Basel).

[50] Chan DSM, Vieira R, Abar L (2023). Postdiagnosis body fatness, weight change and breast cancer prognosis: Global Cancer Update Program (CUP global) systematic literature review and meta-analysis. Int J Cancer.

[51] Ligorio F, Zambelli L, Bottiglieri A (2021). Hormone receptor status influences the impact of body mass index and hyperglycemia on the risk of tumor relapse in early-stage HER2-positive breast cancer patients. Ther Adv Med Oncol.

[52] Fontanella C, Lederer B, Gade S (2015). Impact of body mass index on neoadjuvant treatment outcome: a pooled analysis of eight prospective neoadjuvant breast cancer trials. Breast Cancer Res Treat.

[53] Chen L, Wu F, Chen X (2023). Impact of body mass index in therapeutic response for HER2 positive breast cancer treated with neoadjuvant targeted therapy: a multi-center study and meta-analysis. NPJ Breast Cancer.

[54] Schrijvers JK, McNaughton SA, Beck KL, Kruger R (2016). Exploring the Dietary Patterns of Young New Zealand Women and Associations with BMI and Body Fat. Nutrients.

[55] Kumma WP, Loha E (2023). Dietary patterns and their association with cardiovascular risk factors in Ethiopia: A community-based cross-sectional study. Front Nutr.

[56] Raatz SK, Conrad Z, Johnson LK, Picklo MJ, Jahns L (2017). Relationship of the Reported Intakes of Fat and Fatty Acids to Body Weight in US Adults. Nutrients.

[57] Seargent JM, Yates EA, Gill JH (2004). GW9662, a potent antagonist of PPARgamma, inhibits growth of breast tumour cells and promotes the anticancer effects of the PPARgamma agonist rosiglitazone, independently of PPARgamma activation. Br J Pharmacol.

