# Supplementary material for: PPARG Activation of Fatty Acid Metabolism Drives Resistance to Anti-HER2 Therapies in HER2-Positive Breast Cancer
Source: Int J Biol Sci. 2025 Mar 10;21(6):2396–414. doi: 10.7150/ijbs.99275 (PMC12035890; doi:10.7150/ijbs.99275)
Supplement: Supplementary file 1 — Supplementary figures and tables. [file ijbsv21p2396s1.pdf]

Supplementary table 1
Up-regulated genes expressed in resistant cells

GeneID	FC_PR1vsWT	FC_PR2vsWT	FC_PR3vsWT
ACTG2	4.551433927	4.411335526	4.057526318
CDH5	2.834495377	3.891525259	3.7988458
CPED1	2.807567467	4.289786292	3.54941775
CTTNBP2	3.280557129	3.33054698	3.228525522
CYP4F8	3.387833813	3.783098058	2.594438978
DYNLT3	3.146498872	2.557148692	2.676542776
LOC101926982	3.224415523	3.323267724	2.78837615
LOC105371930	4.438836102	4.321625326	2.571000859
MARCH11	3.015841831	3.551449322	3.078238904
MME	3.261810264	4.685406883	3.23673967
NTSR2	2.707860883	3.137797302	5.742211735
PPARG	2.661302336	3.588735952	2.910098692
RBP2	3.53308382	10.17689763	3.00718889
TAS2R14	2.654582185	3.314289531	3.572075519
TWIST2	3.782976404	6.121123854	5.573750506

Supplementary table 2

The Correlation between PPARG Expression and Clinical Characteristics of Patients

Patient Characteristics		PPARG-High		PPARG-Low		*p* values
		Num	Percentage	Num	Percentage	
Age						
	≤35	0	0	6	5.1282	
	36-45	12	15.7895	17	14.5299	
	46-55	34	44.7368	46	39.3162	0.320
	56-65	24	31.5789	41	35.0427	
	≥66	6	7.8947	7	5.9829	
Local therapy						
	Breast-conserving surgery	3	3.9474	8	6.8376	0.532
	Mastectomy	73	96.0526	109	93.1624	
Histological grade						
	II	31	40.7895	59	50.4274	
	II-III	7	9.2105	7	5.9829	0.253
	III	38	50.0000	48	41.0256	
	Unknown	0	0	3	2.5641	
pT						
	pT1	19	25.0000	27	23.0769	
	pT2	53	69.7368	85	72.6496	0.829
	pT3	4	5.2632	5	4.2735	
pN						
	pN0	37	48.6842	59	50.4274	
	pN1-3	37	48.6842	58	49.5726	0.288
	Unknown	2	2.6316	0	0	
Ki-67						
	≤14%	16	21.0526	23	19.6581	
	＞14%	39	51.3158	59	50.4274	0.942
	Unknown	21	27.6316	35	29.9145	

HR status						
	Negative	60	78.9474	97	82.906	0.571
	Positive	16	21.0526	20	17.094	
Menopause Status						
	No	31	40.7895	43	36.7521	0.650
	Yes	45	59.2105	74	63.2479	

Supplementary table 3

Univariate Cox Regression Analysis of OS in HER2-positive Breast Cancer

Patient Characteristics		*p values*	HR (95%)
Age			
	≤45		
	＞45	0.339	1.799(0.540-5.994)
Local therapy			
	Mastectomy		
	Breast-conserving surgery	0.556	0.556(0.075-4.119)
Histological grade			
	II		
	II-III	0.038	4.543(1.085-19.024)
	III	**0.006**	4.060(1.507-10.939)
	Unknown		
pT			
	pT1		
	pT2	0.163	2.372(0.705-7.984)
	pT3	**0.035**	5.612(1.132-27.812)
pN			
	pN0		
	pN1-3	**0.026**	2.571(1.118-5.915)
	Unknown		
HR status			
	Negative		
	Positive	0.250	1.663(0.699-3.958)
Menopause Status			
	No		
	Yes	0.098	2.160(0.867-5.380)
PPARG expression			
	Low		
	High	**0.028**	2.388(1.096-5.203)

Supplementary table 4

Univariate Cox Regression Analysis of DFS in HER2-positive Breast Cancer

Patient Characteristics		*p values*	HR (95%)
Age			
	≤45		
	＞45	0.910	0.959(0.462-1.991)
Local therapy			
	Mastectomy		
	Breast-conserving surgery	0.474	1.457(0.520-4.083)
Histological grade			
	II		
	II-III	0.600	1.389(0.407-4.744)
	III	0.119	1.633(0.882-3.025)
	Unknown		
pT			
	pT1		
	pT2	0.334	1.463(0.676-3.168)
	pT3	0.055	3.237(0.973-10.766
pN			
	pN0		
	pN1-3	**0.003**	2.575(1.369-4.842)
	Unknown		
HR status			
	Negative		
	Positive	0.099	1.745(0.900-3.382)
Menopause Status			
	No		
	Yes	0.360	1.336(0.719-2.484)
PPARG expression			
	Low		
	High	**0.036**	1.872(1.042-3.363)

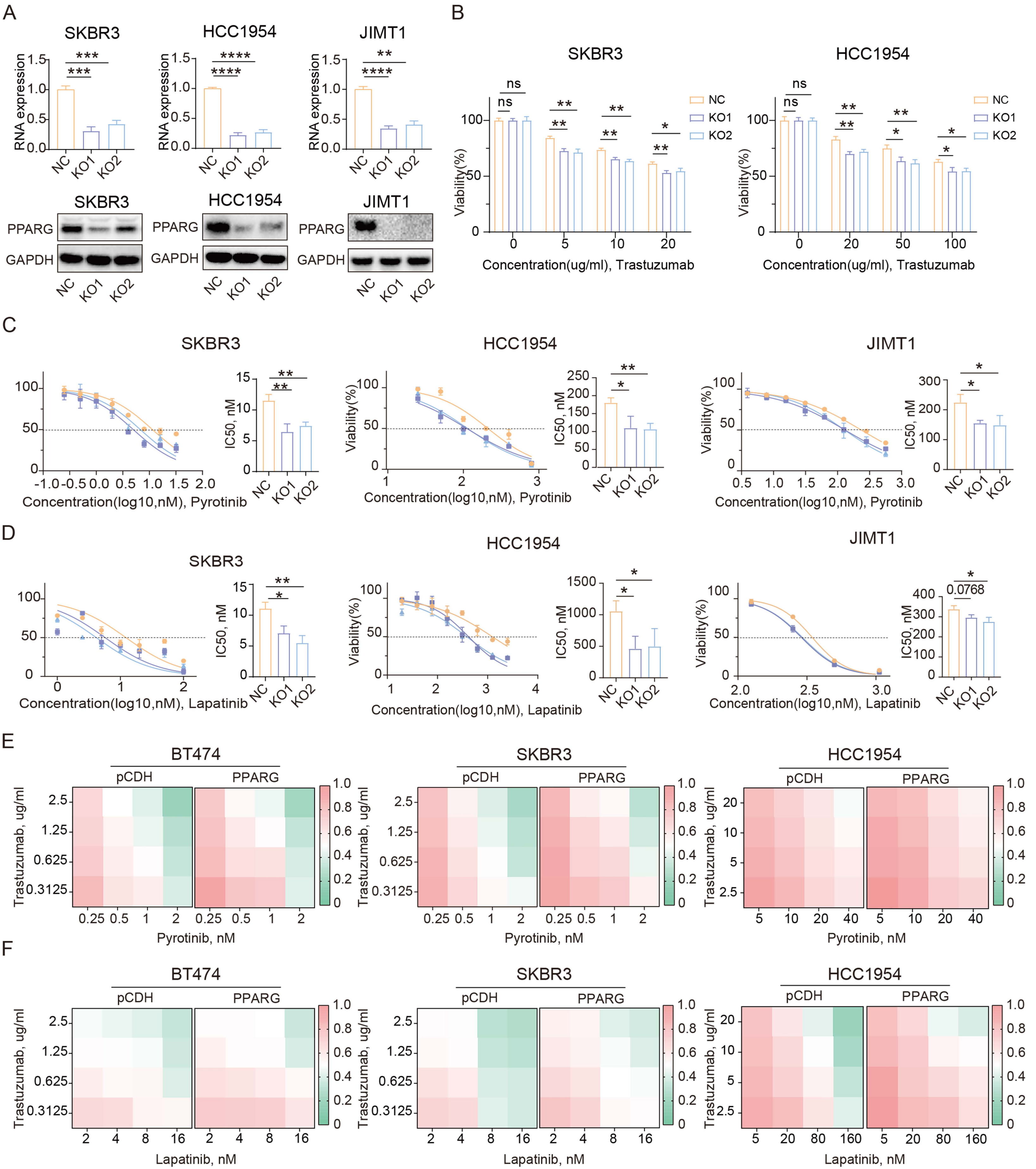

A
SKBR3
HCC1954
JIMT1
RNA expression
NC
KO1
KO2
PPARG
GAPDH
B
SKBR3
HCC1954
NC
KO1
KO2
Viability(%)
Concentration(ug/ml), Trastuzumab
C
Concentration(log10,nM), Pyrotinib
IC50, nM
D
Concentration(log10,nM), Lapatinib
E
BT474
pCDH
PPARG
Trastuzumab, ug/ml
Pyrotinib, nM
F
Lapatinib, nM

Supplementary Figure 1: (A) Expression of PPARG at the RNA and protein level in SKBR3, HCC1954, JIMT1 cells in NC and PPARG knockout groups; (B) Viability of different concentrations of trastuzumab treatment SKBR3, HCC1954 cells with PPARG knockout compared to NC groups; (C) Gradient concentrations of pyrotinib treatment SKBR3, HCC1954, JIMT1 cells with PPARG knockout compared to NC groups were plotted for IC_{50} curves and IC_{50} value bar graphs; (D) Gradient concentrations of lapatinib treatment SKBR3, HCC1954, JIMT1 cells with PPARG knockout compared to NC groups were plotted for IC_{50} curves and IC_{50} value bar graphs; (E) Heat map illustrating the survival rates of BT474, SKBR3, HCC1954 cells with PPARG overexpression and pCDH groups in combination with trastuzumab and pyrotinib treatment; (F) Heat map illustrating the survival rates of BT474, SKBR3, HCC1954 cells with PPARG overexpression and pCDH groups in combination with trastuzumab and lapatinib treatment.

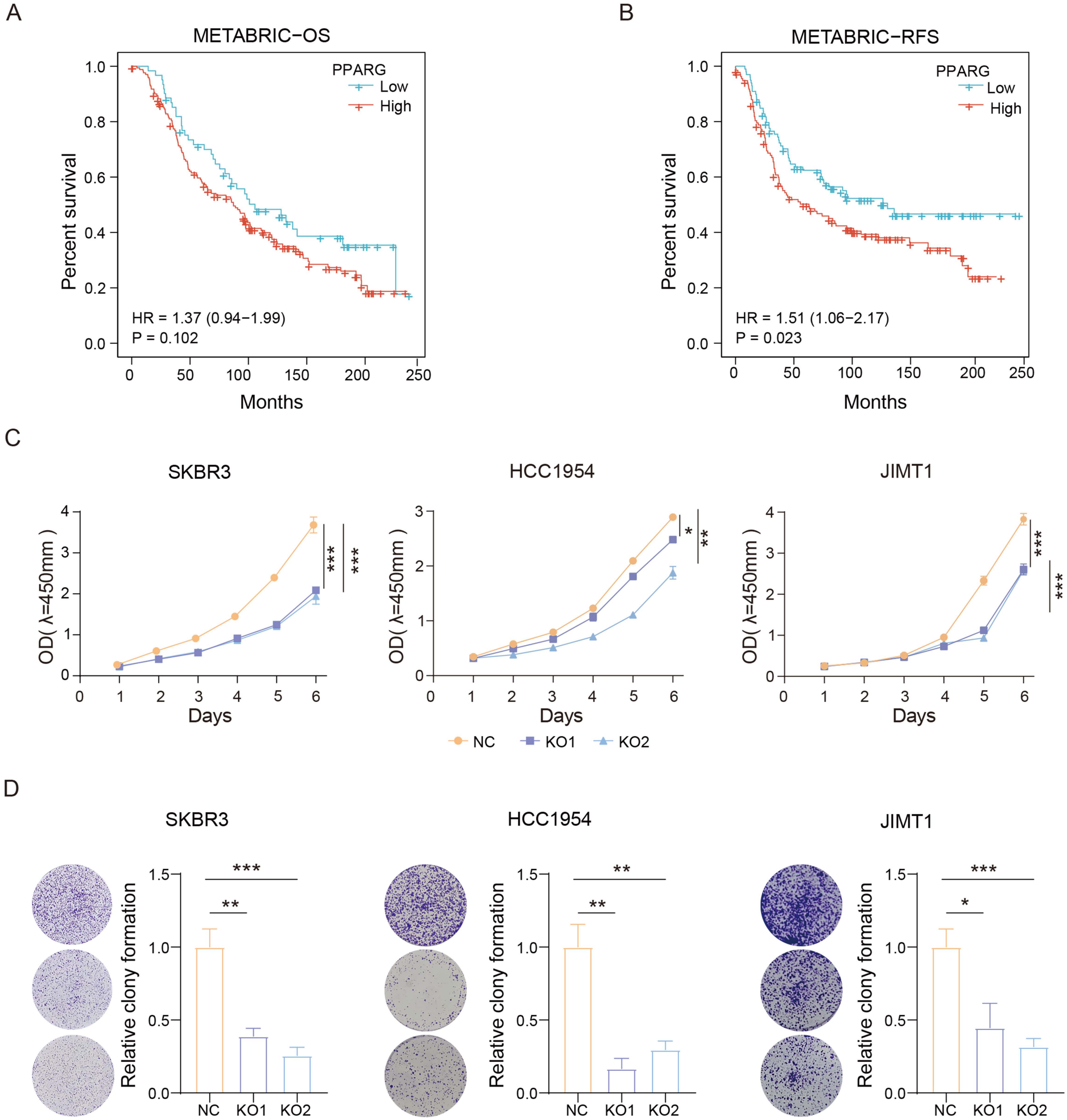

A
METABRIC−OS
PPARG
Low
High
HR = 1.37 (0.94−1.99)
P = 0.102
Percent survival
Months
B
METABRIC−RFS
PPARG
Low
High
HR = 1.51 (1.06−2.17)
P = 0.023
Percent survival
Months
C
SKBR3
HCC1954
JIMT1
OD(λ=450mm)
Days
NC
KO1
KO2
D
SKBR3
HCC1954
JIMT1
Relative clony formation
NC
KO1
KO2

Supplementary Figure 2: Kaplan-Meier analysis of the relationship between PPARG expression levels and (A) OS and (B) DFS in HER2-positive breast cancer patients from METABRIC database; (C) Proliferation curves of SKBR3, HCC1954, and JIMT1 cells PPARG knockout compared to NC groups; (D) Plate colony formation of SKBR3, HCC1954, and JIMT1 cells PPARG knockout compared to NC groups.

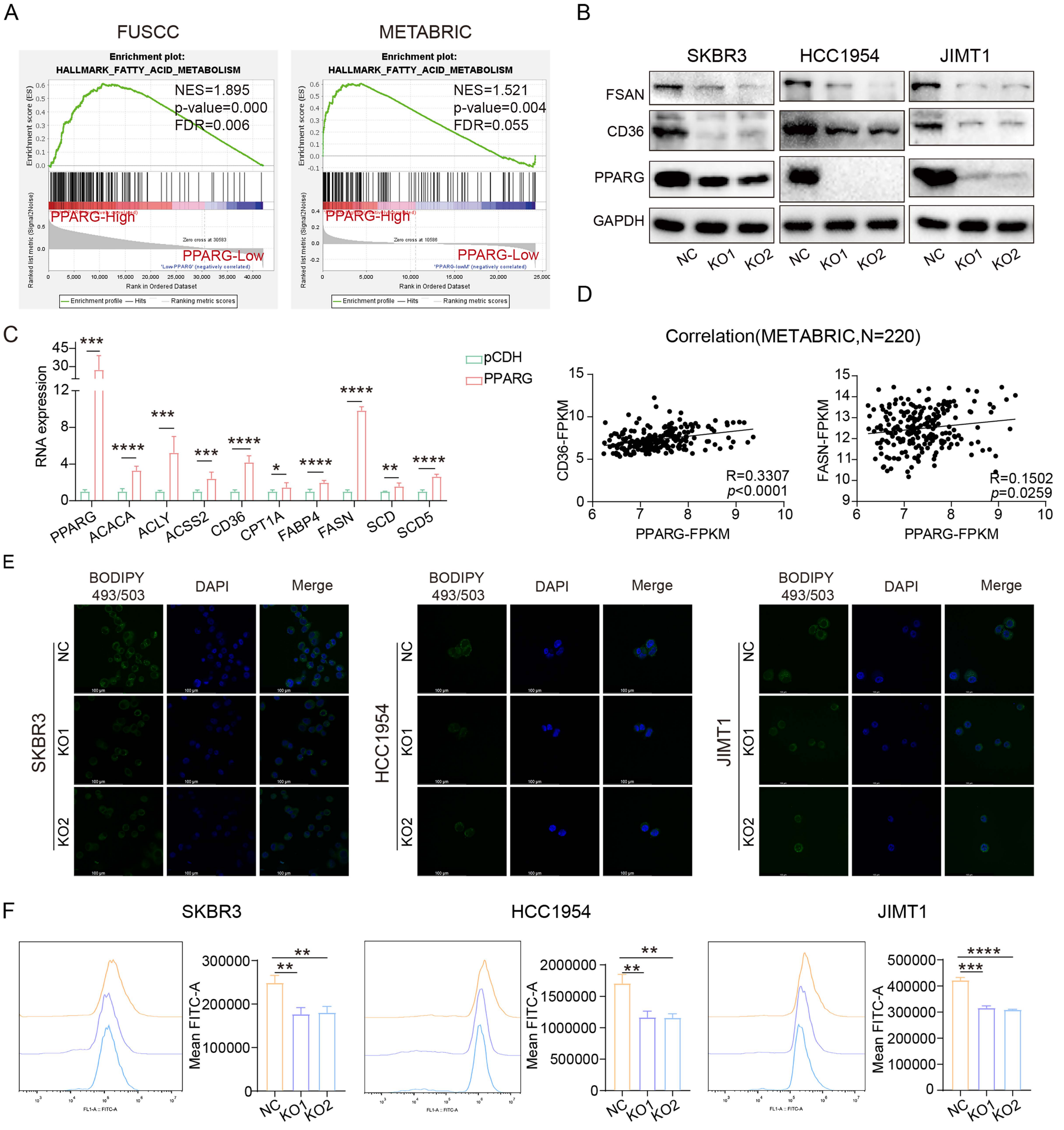
A
FUSCC
METABRIC
Enrichment plot: HALLMARK_FATTY_ACID_METABOLISM
NES=1.895
p-value=0.000
FDR=0.006
NES=1.521
p-value=0.004
FDR=0.055
PPARG-High
PPARG-Low
Enrichment score (ES)
Ranked list metric (Signal2Noise)
Rank in Ordered Dataset
Enrichment profile
Hits
Ranking metric scores
B
SKBR3
HCC1954
JIMT1
FSAN
CD36
PPARG
GAPDH
NC
KO1
KO2
C
RNA expression
pCDH
PPARG
PPARG
ACACA
ACLY
ACSS2
CD36
CPT1A
FABP4
FASN
SCD
SCD5
D
Correlation(METABRIC,N=220)
CD36-FPKM
FASN-FPKM
PPARG-FPKM
R=0.3307
p<0.0001
R=0.1502
p=0.0259
E
BODIPY 493/503
DAPI
Merge
SKBR3
HCC1954
JIMT1
F
SKBR3
HCC1954
JIMT1
Mean FITC-A
FL1-A :: FITC-A

Supplementary Figure 3: (A) GSEA enrichment analysis of the fatty acid metabolism pathway with high and low expression of PPARG from FUSCC and METABRIC database; (B) Western blot of FASN and CD36 in SKBR3, HCC1954, JIMT1 cells with PPARG knockout compared to NC groups; (C) Expression levels of PPARG, ACACA, ACLY, ACSS2, CD36, CPT1A, FABP4, FASN, SCD and SCD5 in PPARG-overexpressing and pCDH group in mice with tumor formation in situ; (D) Correlation analysis between PPARG and CD36, FASN in the METABRIC database; (E) Fatty acid distribution in SKBR3, HCC1954, and JIMT1 cells PPARG knockout compared to NC groups; (F) Fatty acid distribution in SKBR3, HCC1954, and JIMT1 cells PPARG knockout compared to NC groups. ACACA: Acetyl-CoA Carboxylase Alpha; ACSS2: Acyl-CoA Synthetase Short Chain Family Member 2; FABP4: Fatty Acid-Binding Protein 4; SCD5: Stearoyl-CoA Desaturase.

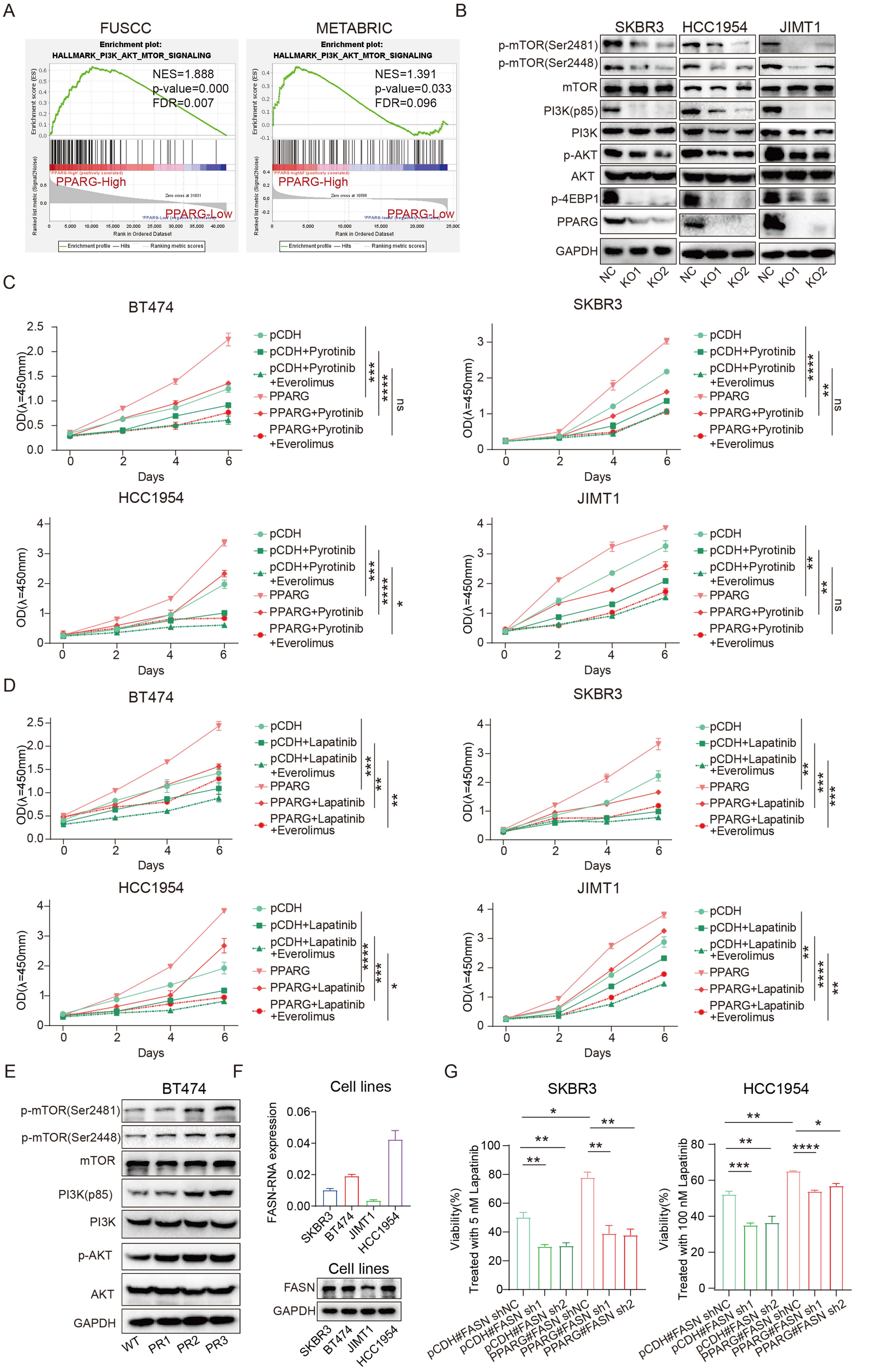

A
FUSCC
Enrichment plot:
HALLMARK_PI3K_AKT_MTOR_SIGNALING
NES=1.888
p-value=0.000
FDR=0.007
PPARG-High
PPARG-Low
METABRIC
Enrichment plot:
HALLMARK_PI3K_AKT_MTOR_SIGNALING
NES=1.391
p-value=0.033
FDR=0.096
PPARG-High
PPARG-Low
Enrichment score (ES)
Ranked list metric (Signal2Noise)
Rank in Ordered Dataset
Enrichment profile
Hits
Ranking metric scores
B
SKBR3
HCC1954
JIMT1
p-mTOR(Ser2481)
p-mTOR(Ser2448)
mTOR
PI3K(p85)
PI3K
p-AKT
AKT
p-4EBP1
PPARG
GAPDH
NC
KO1
KO2
C
BT474
SKBR3
HCC1954
JIMT1
OD(λ=450mm)
Days
pCDH
pCDH+Pyrotinib
pCDH+Pyrotinib +Everolimus
PPARG
PPARG+Pyrotinib
PPARG+Pyrotinib +Everolimus
D
BT474
SKBR3
HCC1954
JIMT1
OD(λ=450mm)
Days
pCDH
pCDH+Lapatinib
pCDH+Lapatinib +Everolimus
PPARG
PPARG+Lapatinib
PPARG+Lapatinib +Everolimus
E
BT474
p-mTOR(Ser2481)
p-mTOR(Ser2448)
mTOR
PI3K(p85)
PI3K
p-AKT
AKT
GAPDH
WT
PR1
PR2
PR3
F
Cell lines
FASN-RNA expression
SKBR3
BT474
JIMT1
HCC1954
Cell lines
FASN
GAPDH
SKBR3
BT474
JIMT1
HCC1954
G
SKBR3
Viability(%)
Treated with 5 nM Lapatinib
HCC1954
Viability(%)
Treated with 100 nM Lapatinib
pCDH#FASN shNC
pCDH#FASN sh1
pCDH#FASN sh2
PPARG#FASN shNC
PPARG#FASN sh1
PPARG#FASN sh2

Supplementary Figure 4: (A) GSEA analysis of the PI3K/Akt/mTOR signaling pathway in FUSCC and METABRIC databases (PPARG-high vs PPARG-low); (B) Expression levels of PI3K/Akt/mTOR signaling pathway proteins in SKBR3, HCC1954 and JIMT1 cells with PPARG knockout compared to NC groups; (C) Proliferation curves of BT474, SKBR3, HCC1954 and JIMT1 cells PPARG-overexpressing group and pCDH group treated with or without Pyrotinib/ everolimus; (D) Proliferation curves of BT474, SKBR3, HCC1954 and JIMT1 cells PPARG-overexpressing group and pCDH group treated with or without Lapatinib/ everolimus; (E) Expression levels of PI3K/Akt/mTOR signaling pathway proteins in BT474 WT and PR cells; (F) Expression of FASN at the RNA and protein level in four HER2-positive breast cancer cell lines (SKBR3, BT474, JIMT1, HCC1954 cells); (G) Viability of SKBR3 and HCC1954 cells treated with lapatinib.

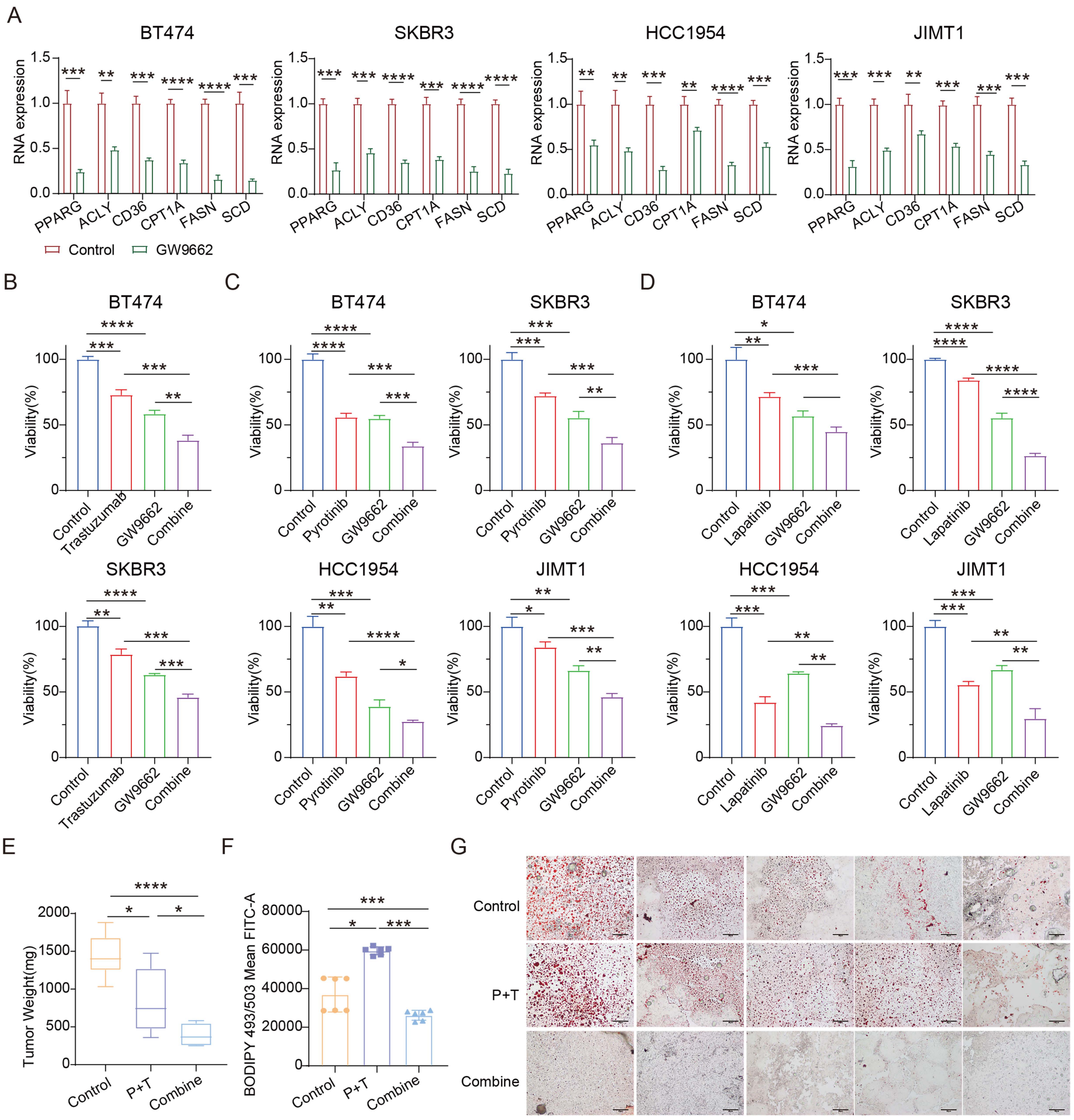

A
BT474
SKBR3
HCC1954
JIMT1
RNA expression
PPARG
ACLY
CD36
CPT1A
FASN
SCD
Control
GW9662
B
C
D
Viability(%)
Control
Trastuzumab
Pyrotinib
Lapatinib
GW9662
Combine
E
Tumor Weight(mg)
P+T
F
BODIPY 493/503 Mean FITC-A
G
Control
P+T
Combine

Supplementary Figure 5: (A) GW9662 treatment effects on the expression of PPARG, ACLY, CD36, CPT1A, FASN, and SCD in BT474, SKBR3, HCC1954, and JIMT1 cells; (B) Cell viability when BT474 and SKBR3 cells were treated with GW9662 and trastuzumab alone or in combination; (C) Cell viability when BT474, SKBR3, HCC1954, JIMT1 cells were treated with GW9662 and pyrotinib alone or in combination; (D) Cell viability when BT474, SKBR3, HCC1954, JIMT1 cells were treated with GW9662 and lapatinib alone or in combination; (E) Mass box diagram of in situ tumors in Control, P+T, and Combine groups; (F) Lipid fluorescence values of in situ tumors in Control, P+T, and Combine groups; (G) Oil Red staining images of in situ tumors in Control, P+T, and Combine group mice.

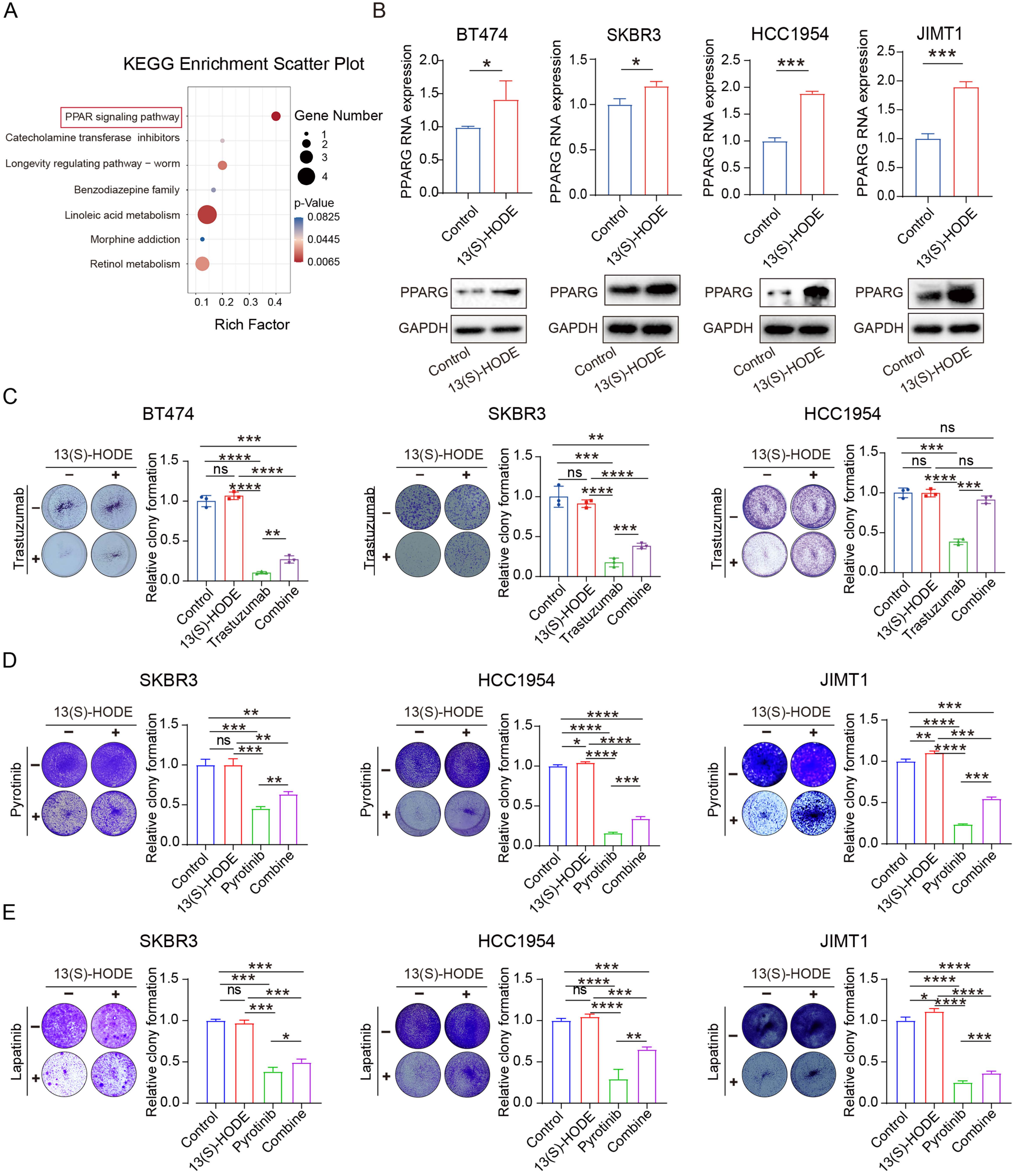

A
KEGG Enrichment Scatter Plot
PPAR signaling pathway
Catecholamine transferase inhibitors
Longevity regulating pathway − worm
Benzodiazepine family
Linoleic acid metabolism
Morphine addiction
Retinol metabolism
Gene Number
p-Value
Rich Factor
B
BT474
SKBR3
HCC1954
JIMT1
PPARG RNA expression
Control
13(S)-HODE
PPARG
GAPDH
C
D
E
13(S)-HODE
Trastuzumab
Pyrotinib
Lapatinib
Relative clony formation
Combine

Supplementary Figure 6: (A) Enrichment of the PPAR signaling pathway in the non-pCR group; (B) RNA and protein expression of PPARG after treatment with 13(S)-HODE in BT474, SKBR3, HCC1954, JIMT1 cells; (C) Plate clone formation of BT474, SKBR3, and HCC1954 cells with or without 13(S)-HODE and trastuzumab treatment; (D Plate clone formation of SKBR3, HCC1954 and JIMT1 cells with or without 13(S)-HODE and pyrotinib treatment; (E) Plate clone formation of SKBR3, HCC1954 and JIMT1 cells with or without 13(S)-HODE and lapatinib treatment.
